# Effect of Self‐Assembling Peptide on White Spot Lesions in Orthodontic Patients: A Systematic Review and Meta‐Analysis

**DOI:** 10.1002/cre2.70321

**Published:** 2026-02-26

**Authors:** Sheema Shakir, Farhana Umer, Qasim Khalid, Khurram Shahzad, Umar Hussain, Samrina Mohammad, Hamna Omair, Alessandra Campobasso, Waqas Naseem, Junad Khan

**Affiliations:** ^1^ Department of Prosthodontics Khyber College of Dentistry Peshawar Pakistan; ^2^ CMH Medical College and Institute of Dentistry Lahore Pakistan; ^3^ Orofacial Pain and TMD, Eastman Institute of Oral Health University of Rochester Rochester New York USA; ^4^ Orthodontics, Avicenna Medical and Dental College Lahore Pakistan; ^5^ Orthodontics, Saidu College of Dentistry Saidu Sharif Pakistan; ^6^ Oral Pathology Department Khyber College of Dentistry Peshawar Pakistan; ^7^ PGR, CMH Lahore Medical College and Institute of Dentistry Lahore Pakistan; ^8^ Department of Clinical and Experimental Medicine University of Foggia Foggia Italy; ^9^ Department of Health Sciences University of Debrecen Debrecen Hungary

**Keywords:** meta‐analysis, orthodontic patients, self‐assembling peptide, systematic review, white spot lesion

## Abstract

**Background:**

**Objective**: To evaluate the effect of self‐assembling peptide (SAP) on white spot lesions (WSLs) in orthodontic patients with fixed appliances.

**Methods:**

**Eligibility Criteria**: Randomized and non‐randomized clinical studies, as well as in vitro studies, comparing SAP with fluoride or no treatment for WSLs in orthodontic patients were included. Outcomes assessed were WSL score, lesion size, or fluorescence loss. Case reports, case series, animal studies, and narrative reviews were excluded.

**Information Sources:** Comprehensive literature search of six databases was conducted up to May 16, 2025.

**Risk of Bias**: Risk of bias was assessed using tools appropriate to each study design: RoB‐2 for randomized controlled trials, ROBINS‐I for non‐randomized studies, and QUIN for in vitro studies.

**Synthesis of Results:** Random effects meta‐ analyses using standardized mean differences (SMDs) and their 95% confidence intervals (CIs) were performed, followed by subgroup, sensitivity analyses, and assessment of the quality of evidence was done using GRADE approach.

**Results:**

**Included Studies**: Eight studies (five randomized, two non‐randomized prospective, one in vitro) involving 395 participants were included.

**Synthesis of Results:** Meta‐analysis of eight studies showed a significant reduction in severity of WSLs with SAP compared to fluoride or control (SMD = −1.68; 95% CI: −2.71 to −0.67; *p* = 0.005), but with high heterogeneity (*I* ^2^ = 85.4%) and wide prediction interval (−4.20 to 0.82). Evidence certainty was very low due to risk of bias, inconsistency, imprecision, and indirectness.

**Discussion:**

**Limitations of Evidence:** The study is limited by the inclusion of less number of studies, one in‐vitro study, and some non‐randomized trials.

**Interpretation/Conclusion**: It can be concluded that very low‐level evidence is available that SAP can reduce the severity of WSLs associated with fixed orthodontic treatment more effectively than topical fluoride or no treatment.

**Registration**: **PROPSERO Registration Number:** (CRD420251112657).

**Abbreviations**
ACP: amorphous calcium phosphate
CCT: controlled clinical trial
CI: confidence interval
CPP‐ACP: casein phosphopeptide‐amorphous calcium phosphate
CPP‐ACPF: casein phosphopeptide‐amorphous calcium phosphate with fluoride
GRADE: grading of recommendations assessment, development and evaluation
*I* ^2^
: inconsistency index (heterogeneity measure)
ICC: intra‐cluster correlation coefficient
OPD: outpatient department
PICOS: population, intervention, comparison, outcomes, and study design
PRISMA: Preferred Reporting Items for Systematic Reviews and Meta‐Analyses
QUIN: quality assessment tool for in vitro studies
RCT: randomized controlled trial
ROBINS‐I: risk of bias in non‐randomized studies—of interventions
RoB 2: revised cochrane risk of bias tool for randomized trials
SAP: self‐assembling peptide
SMD: standardized mean difference
VAS: visual analogue scale
WSL: white spot lesion

## 1 Introduction

Dental caries is the most common dental issue worldwide (Ozdemir 2013). White spot lesions (WSLs) associated with fixed appliance therapy, are early enamel demineralizations that appear as chalky white areas around orthodontic attachments due to prolonged plaque accumulation that leads to localized acidic attacks on the enamel (Lazar et al. 2023). These are among the most common adverse effects of fixed orthodontic treatment (Shankarappa et al. 2024). WSLs affect approximately 25%–50% of patients undergoing fixed orthodontic therapy, with higher incidence in adolescents due to variable oral hygiene practices and dietary habits (Höchli et al. 2017). Risk factors include poor plaque control, high sugar intake, duration of appliance therapy, and individual salivary flow rates (Srivastava et al. 2013). Beyond their esthetic concern, WSLs are early signs of enamel demineralization that can progress to cavitated carious lesions if left untreated (Puleio et al. 2022).

Due to the network architecture of orthodontic fixed appliances, maintaining optimal oral hygiene becomes challenging, necessitating the use of some therapeutic agents to prevent the development of WSLs (Hussain et al. 2025). Several therapeutic strategies have been introduced for preventing and managing the WSLs, including fluoride applications (Nascimento et al. 2016), casein phosphopeptide‐amorphous calcium phosphate (CPP‐ACP) (Baccolini et al. 2025), and resin infiltration (Borges et al. 2017). However, many of these modalities are limited by their superficial remineralization potential, failing to reverse deeper subsurface lesions effectively. Topical fluoride application remains the gold standard for the prevention of WSLs during orthodontic therapy (Sonesson and Twetman 2023). Despite its effectiveness, fluoride has some limitations, like its inability to completely reverse early WSLs, as it depends on residual enamel crystals and the availability of calcium and phosphate ions for remineralization (Soares et al. 2017). These limitations of fluoride have led to the development of alternative bioactive strategies, such as the CPP‐ACP system that stabilizes calcium and phosphate ions in the oral environment. When combined with fluoride, forming CPP‐ACPF, which demonstrates superior preventive efficacy compared to CPP‐ACP alone (Oliveira et al. 2016). Though CPP‐ACP and CPP‐ACPF have shown some improvement in enamel hardness and mineral content but are less effective in deep subsurface lesions (Erramshetty et al. 2024). Resin infiltration offers aesthetic improvement by masking lesion opacity, but it does not restore the mineral content of enamel (Anand et al. 2019).

Self‐assembling peptides (SAPs) have demonstrated enhanced efficacy in the management of WSLs compared to conventional remineralizing agents such as fluoride and CPP‐ACP. Unlike fluoride and CPP‐ACP, SAPs act by penetrating the lesion body and forming a biomimetic three‐dimensional scaffold (Brunton et al. 2013). This scaffold promotes *de novo* hydroxyapatite formation through a process of guided enamel regeneration, independent of residual mineral content. As a result, SAPs result in deeper and more remineralization of early carious lesions (Ghaly et al. 2023). Emerging clinical evidence, particularly in orthodontic populations (Ghaly et al. 2023; Ali, Abo Elezz et al. 2022; Gohar et al. 2023; Welk et al. 2020), supports the superior performance of SAPs in reducing lesion depth and severity, with significant aesthetic and preventive benefits over standard fluoride‐ or CPP‐ACP‐based therapies.

Two previous systematic reviews evaluated the efficacy of SAPs on WSLs in non‐orthodontic populations. Wierichs et al. (2021) included seven randomized controlled trials, and Rathore et al. (2023) included eight trials assessing SAPs with or without fluoride. While both reviews reported some improvement in lesion appearance, their included studies were conducted in non‐orthodontic subjects, where much higher risk exist for WSLs development in patients undergoing fixed appliance therapy. WSLs pose distinct clinical challenges in orthodontic patients, primarily due to increased plaque retention around fixed appliances. However, the effectiveness of these peptides in orthodontic patients remains unclear. This systematic review and meta‐analysis aims to address this gap by systematically evaluating the evidence on SAPs for the prevention and treatment of WSLs associated with fixed orthodontic appliances. Our hypothesis was SAPs are more effective than fluoride or no treatment in reducing the incidence and severity of WSLs in orthodontic patients.

## 2 Methodology

### 2.1 Registration and Protocol

This systematic review and meta‐analysis was performed following the guidelines outlined in the Cochrane Handbook (Higgins et al. 2022), and reported according to the Preferred Reporting Items for Systematic Reviews and Meta‐Analyses (PRISMA) 2020 statement (Liberati et al. 2009). The study was registered in the International Prospective Register of Systematic Reviews (CRD420251112657).

### 2.2 Eligibility Criteria

The eligibility criteria were based on the PICOS format (Population, Intervention, Comparison, Outcomes, and Study Design); **P:** Orthodontic patients of any age and gender with WSLs associated with fixed orthodontic appliances; **I:** SAPs; **C:** Fluoride varnish, CPP‐ACP, placebo, untreated controls, or other standard care interventions; **O:** Score, size, or frequency of WSLs; and **S:** Randomized controlled trials (RCTs), non‐randomized clinical trials, prospective and retrospective cohort studies, as well as in vitro studies simulating orthodontic conditions. Excluded were case series, case reports, animal studies, and narrative reviews.

### 2.3 Information Sources and Search Strategy

An unrestricted literature search was conducted across six major electronic databases: PubMed, Cochrane CENTRAL, Scopus, LILACS, Web of Science, and Google Scholar (first 100 results only). In addition to these databases, gray literature sources such as ProQuest were also searched for unpublished or non‐peer‐reviewed work. The search was performed using a predefined search strategy, as outlined in Table S1. The search strategy was designed to capture all publications from the earliest available records up to May 16, 2025, without applying any restrictions on the publication date, language, or type of study. The reference lists of all included eligible studies and previously published systematic reviews were manually screened to identify any additional relevant studies that may have been missed in the initial electronic database search. Two authors (A.C. and J.K.) performed the search independently and any discrepancy were resolved through discussion.

### 2.4 Selection Process

The studies identified in the database search were saved in a Microsoft Excel 2016 sheet, where the titles were sorted in ascending order and duplicates were automatically highlighted in red font using an Excel function. A new adjacent column was added to manually identify and remove any duplicates. The titles and abstracts of the studies were then assessed against the eligibility criteria to determine relevance. Full texts of the studies that met the initial screening criteria were retrieved and reviewed in detail. The study selection was done independently by two authors (U.M. and R.S.), and any discrepancies were resolved through discussion with a third author (A.C.).

### 2.5 Risk of Bias

The risk of bias in included studies was assessed using appropriate tools based on study design. The revised Cochrane Risk of Bias Tool for RCTs (RoB 2) (Flemyng et al. 2023) was used to evaluate the RCTs. For non‐randomized studies, the Risk of Bias in Non‐randomized Studies—of Interventions (ROBINS‐I) (Jüni et al. 2016) tool was utilized. To assess the risk of bias in in vitro studies, the Quality Assessment Tool for In Vitro Studies (QUIN) (Sheth et al. 2024) was applied.

### 2.6 Effective Sample Size

Some studies reported a clustered split‐mouth or pre‐post design to compare the quantification/score of WSLs between control and SAP groups. To account for clustering, the design effect was applied using the formula: 1 + (*M* – 1) × ICC, where *M* represents the average cluster size (approximately 12 teeth per cluster) and the intra‐cluster correlation coefficient (ICC) was assumed to be 0.2, as suggested by Masood et al. (2015). For continuous outcomes, only the sample size was adjusted, while the means and standard deviations remained unchanged (Higgins et al. 2019).

### 2.7 Effect Measures and Synthesis Measures

Data analyses were conducted using R software (version 4.3.3). Data synthesis was performed when at least two studies reported data for the outcome variables of interest. Due to clinical heterogeneity—such as differences in ethnicity, dispensing methods and concentrations of SAP or comparators, variations in follow‐up duration, study design, and patient age—a random‐effects model was employed to estimate the average of the distribution of true effects (Papageorgiou 2014).

Absolute heterogeneity (tau^2^) was estimated using the restricted maximum likelihood approach, due to its greater reliability (Langan et al. 2019). The standardized mean difference (SMD) was used to estimate pooled effects, as the included studies reported outcome effect sizes using different measures (e.g., various scales for WSL scores, fluorescence loss, color change, and/or mineral content). Hartung–Knapp adjustments were applied to the pooled estimates to reduce potential underestimation caused by small study effects. Between‐study heterogeneity was assessed by examining forest plots, estimating tau^2^, and calculating the *I* ^2^ statistic (relative heterogeneity) (Higgins et al. 2003). To improve the interpretation of random‐effects meta‐analyses, 95% prediction intervals were calculated for meta‐analyses involving three or more studies. These intervals accounted for heterogeneity in treatment effects and provided a range of possible outcomes for future comparable clinical trials (Page et al. 2021). Correction for small sample sizes was applied using Hedges' g to yield more accurate effect size estimates (Taylor and Alanazi 2023).

## 3 Results

### 3.1 Study Searches

A systematic search was conducted across five major databases. The search yielded 203 records from PubMed, 178 from Scopus, 133 from Web of Science, 135 from the Cochrane CENTRAL (1 review and 134 trials), and 9 from LILACS. Full search terms and database‐specific strategies are detailed in Table S1.

### 3.2 Study Selection

**Figure 1:**
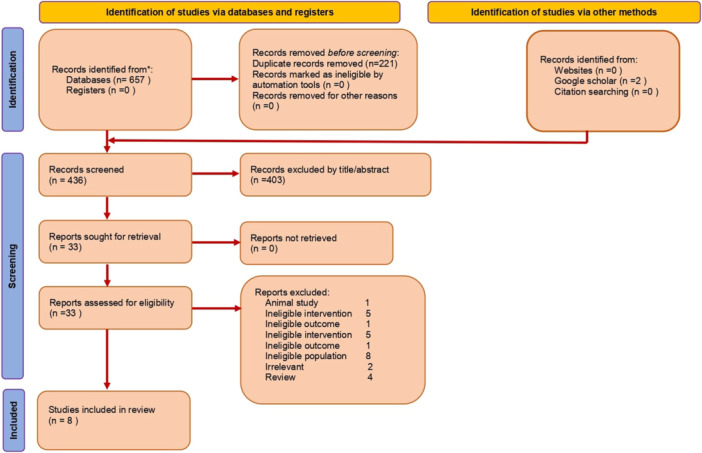
PRISMA flowchart of the study selection process.

A total of 659 records were identified through database and manual searches, comprising 657 records from electronic databases and 2 from Google Scholar. Following the removal of 221 duplicates, 436 records remained for title and abstract screening. Of these, 403 were excluded based on irrelevance or failure to meet the inclusion criteria. Full‐text articles were obtained for 33 records and assessed for eligibility. Of the 33 full‐text articles assessed, 25 were excluded for the following reasons: animal study (*n* = 1), ineligible intervention (*n* = 5), ineligible outcome (*n* = 2), ineligible population (*n* = 8), irrelevant content (*n* = 2), and review articles (*n* = 4). A total of eight studies were deemed eligible and included in the final review (Figure 1).

### 3.3 Study Characteristics

The review included eight clinical studies of which five were RCTs, two prospective non‐randomized studies, and one was in vitro study. The studies were conducted in Egypt, Germany, Turkey, and involved a total of 395 participants, with age ranging from 12 to 25 years. Sample sizes varied from 14 to 108 participants. Among the 121 participants from three studies that reported gender, 47.9% were males. Intervention groups received SAP P11‐4 either alone or in combination with fluoride or amorphous calcium phosphate (ACP), while comparators included fluoride varnish, ACP varnish, or no treatment. Most studies had follow‐up durations ranging from 6 weeks to 6 months. WSLs were assessed using a variety of methods, including DIAGNOdent, quantitative light‐induced fluorescence (QLF), digital x‐ray analysis, impedance scaling, and Vita Easyshade colorimetry. Commonly reported outcomes included WSL scores, lesion area, enamel fluorescence loss (ΔF), lesion volume (ΔQ), surface microhardness, color changes (ΔE*, L* value), and salivary pH (Table 1).

**Table 1:** Characteristics of the included studies.

Study	Design; country	Participants (M/F); mean age (years)	Interventions	Follow‐up time	Method to record WSL/scale	Outcomes measured
Abdel Aziz and Marei (2016) and Page et al. (2021)	Prospective clinical study; Egypt	14(NR);12–22	E: post trt with SAP P11‐4 C: Pre trt	3 mo	Software analyzing digital x‐ray	‐ Enamel mineral content change (%) ‐ Salivary pH change
Welk et al. (2020) and Brunton et al. (2013)	RCT (split‐mouth); Germany	23 (13/10); 15.4	E: SAP P_11_‐4 C: Control	1.5, 3, 6 mo	Impedance scale	‐ WSL score ‐ Lesion area (mm^2^)
Gohar et al. (2023) and Anand et al. (2019)	RCT; Egypt	58 (25/33); ~21	E1: SAP var (*n* = 29) C: F^‐^ var (*n* = 29)	3, 6 mo	DIAGNOdent/ICDAS	‐ WSL score
Jablonski‐Momeni et al. (2020) and Langan et al. (2019)	RCT; Germany	108 Human teeth (NR)	CI: No trt (*n* = 60) E1: Fluoride var (*n* = 60) E2: SAP P11‐4 + Fluoride var (*n* = 60)	7, 30 days	QLF	‐ Fluorescence loss
Güven et al. (2024) and Taylor and Alanazi (2023)	Prospective clinical study; Turkey	32 (NR); 13.91	C: Fluoride var (*n* = 12) E1: SAP P11‐4 (*n* = 10) E2: combined SAP P11‐4 + Fluoride var (*n* = 10)	6 weeks, 3, 6 mo	DIAGNOdent/QLF	‐ ΔF (fluorescence loss %) ‐ ΔQ (lesion volume % × mm^2^) ‐ Lesion area (mm^2^) ‐ Color change (ΔE*)
Ghaly et al. (2023) and Oliveira et al. (2016)	In‐vitro; Egypt	80 human teeth (extracted premolars)	E1: SAP P11‐4 (*n* = 20) E2: CPP‐ACP (*n* = 20) E3: Fluoride var (n = 20) C: No trt (*n* = 20)	2, 4 weeks	Surface microhardness test	‐ Surface microhardness —
Ali, Abo Elezz et al.(2022), Ali, Ismail et al. (2022) and Erramshetty et al. (2024)	RCT; Egypt	40 (NR); 16–25	C: Control (*n* = 10) E1: SAP P11‐4 (*n* = 10) E2: ACP var (*n* = 10) E3: Combination (SAP + ACP) (*n* = 10)	1, 3, 6 mo	DIAGNOdent	‐ Fluorescence loss
Riad et al. (2020) and Higgins et al. (2003)	RCT (split‐mouth); Egypt	40 (20/20):12–25	E: SAP P11‐4 (*n* = 40) C: = Fluoride Var (*n* = 40)	3, 6 mo	Vita Easyshade (L* value)	‐ Color change of WSL (L* value)

Abbreviations: ACP, amorphous calcium phosphate; C, control; E, experimental; ICDAS, International Caries Detection and Assessment System; mo, months; NRS, non‐randomized study; QLF, Quantitative Light‐induced Fluorescence; SAP P11‐4, self‐assembling peptide P11‐4; trt, treatment; WSL, white spot lesion.

### 3.4 Risk of Bias Assessment

**Figure 2:**
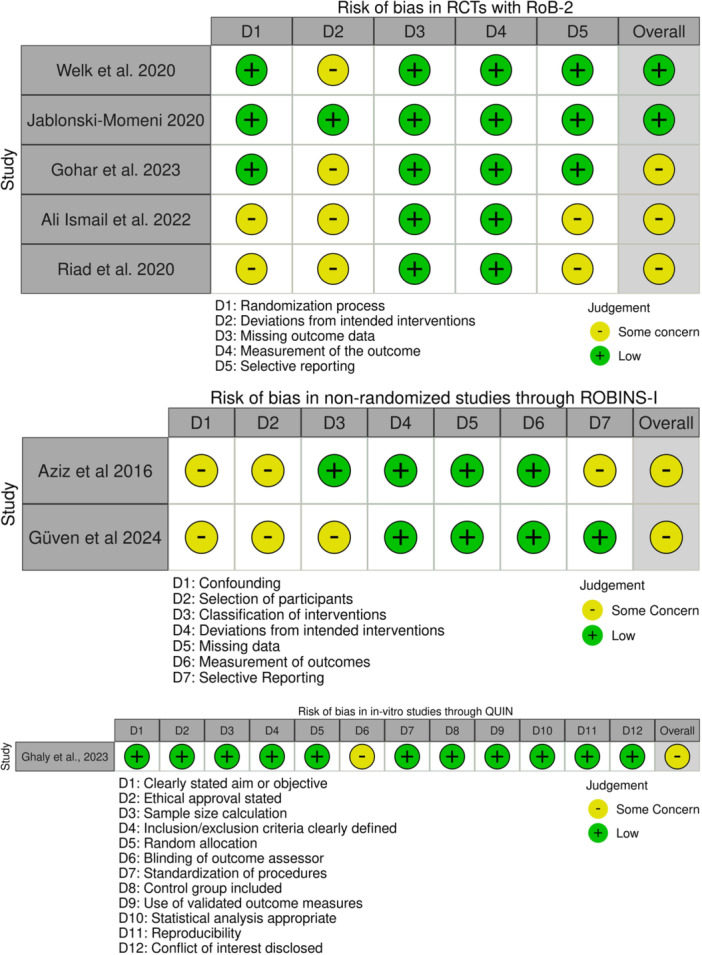
Risk of bias in the included studies.

Risk of bias was assessed using RoB‐2 for RCTs, ROBINS‐I for non‐randomized studies, and QUIN for the in vitro study. Among the five RCTs, two (Welk et al. 2020; Jablonski‐Momeni et al. 2020) showed low risk of bias, while three (Ali, Abo Elezz et al. 2022; Gohar et al. 2023; Riad et al. 2020) had some concerns, primarily related to randomization, deviations from intended interventions, and selective reporting. Both non‐randomized studies (Abdel Aziz and Marei 2016; Güven et al. 2024) were judged to have moderate risk of bias, mainly due to confounding and participant selection. The in vitro study (Ghaly et al. 2023) had a moderate risk of bias, with concerns in blinding and reproducibility despite low risk in most domains (Figure 2).

### 3.5 Efficacy of Self‐Assembling Peptides

**Figure 3:**
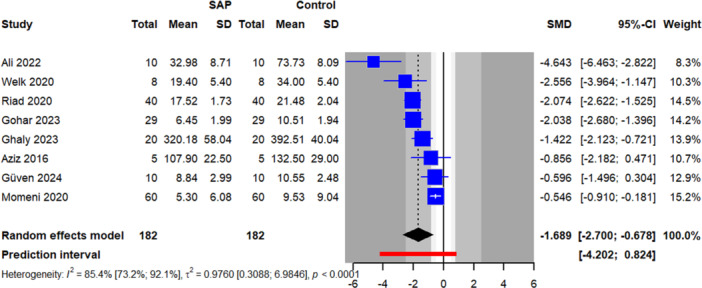
Forest plot for comparing WSL quantification between self‐assembling peptide and fluoride or control groups.

The meta‐analysis of eight studies comparing WSLs quantification between SAPs and fluoride or no treatment showed a statistically significant effect in favor of the peptide, with an SMD of −1.68 (95% CI −2.71 to −0.67, *p* = 0.005). Heterogeneity was substantial (*I* ^2^ = 85.4%, 95% CI 73.2%–92.1%), with between‐study variance (*τ*²) of 0.97 (95% CI = 0.31–6.98). The 95% prediction interval ranged from −4.20 to 0.82 (Table 2 and Figure 3).

**Table 2:** Results of meta‐analyses (≥ 2 studies) comparing WSL quantification between self‐assembling peptide and fluoride or control groups.

Outcome	*n*	SMD [95% CI]	*p*	*τ* ^2^ [95% CI]	*I* ^2^ [95% CI]	95% prediction
WSL quantification	8	−1.68 [−2.71; −0.67]	0.005	0.97 [0.31; 6.98]	85.4% [73.2%; 92.1%]	−4.20; 0.82

Abbreviations: CI, confidence interval; *n*, number of studies; *p*, *p*‐value; SMD, standardized mean difference.

### 3.6 Sources of Heterogeneity–Subgroup Analysis

Sensitivity analyses for WSLs severity comparing SAPs to fluoride or no treatment showed consistent results across most subgroups (Table 3). The overall effect remained significant (SMD = −1.69, 95% CI = −2.70 to −0.68). No significant subgroup differences were found based on comparator type (*p* = 0.79), ethnicity (*p* = 0.74), outcome measure (*p* = 0.21), or risk of bias (*p* = 0.74). However, a significant difference was observed by study design (*p* = 0.011), where RCTs showed a larger effect (SMD = −2.18, 95% CI = −3.92 to −0.45) compared to non‐randomized studies (SMD = −0.68, 95% CI = −2.21 to 0.85). The in vitro study also reported a large effect (SMD = −1.42, 95% CI = −2.12 to −0.72). The observed treatment effect was generally robust across subgroups (Table 3 and Figure S2).

**Table 3:** Sensitivity analyses for meta‐analyses with at least 8 included studies comparing WSL severity between self‐assembling peptide and control/fluoride groups.

Outcome	Predictor	Group/subgroup	*n*	SMD [95% CI]	*p* *
WSL severity	Comparator	Original analysis	8	−1.69 [−2.70; −0.68]	0.79
		No treatment	5	−1.85 [−3.81; 0.11]	
		Fluoride	3	−1.63 [−3.65; 0.40]	
	Study design	Original analysis	8	−1.69 [−2.70; −0.68]	0.011
		NRS	2	−0.68 [−2.21; 0.85]	
		RCT	5	−2.18 [−3.92; −0.45]	
		In vitro	1	−1.42 [−2.12; −0.72]	
	Ethnicity	Original analysis	8	−1.69 [−2.70; −0.68]	0.74
		Asian	6	−1.80 [−3.14; −0.47]	
		European	2	−1.43 [−14.11; 11.25]	
	Outcome measure	Original analysis	8	−1.69 [−2.70; −0.68]	0.21
		Mineral content	1	−0.86 [−2.18; 0.47]	
		WSL score	2	−2.13 [−4.61; 0.35]	
		Fluorescence loss	3	−1.80 [−7.49; 3.89]	
		Color change score	1	−2.07 [−2.62; −1.53]	
		Microhardness	1	−1.42 [−2.12; −0.72]	
	Risk of bias	Original analysis	8	−1.69 [−2.70; −0.68]	0.74
		Some concern	6	−1.80 [−3.14; −0.47]	
		Low	2	−1.43 [−14.11; 11.25]	

* *p*‐values refer to subgroup difference tests based on random‐effects model; *n*, number of studies.

### 3.7 Sources of Heterogeneity–Sensitivity Analysis

Sensitivity analysis was conducted by excluding one in‐vitro study from the pooled effect, and the results remained robust: overall pooled effect (SMD = −1.68[−2.70; −0.67) versus without the in vitro study (SMD = −1.75[−2.99; −0.53]).

### 3.8 Publication Bias

**Figure 4:**
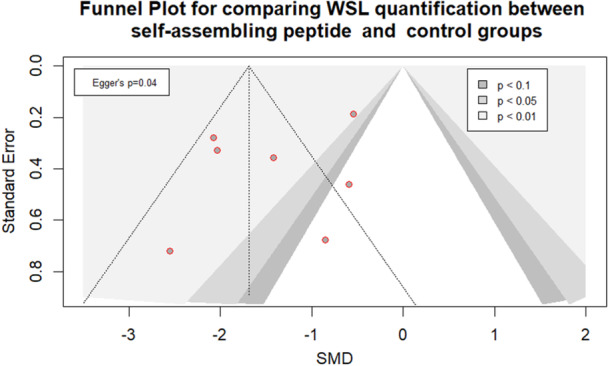
Funnel plot for comparing WSL quantification between self‐assembling peptide and control groups.

Visual inspection of the contour‐enhanced funnel plot suggested asymmetry, with a relative absence of studies on the right‐hand side of the plot (Figure 4). This observation was statistically supported by Egger's regression test (*p* = 0.04), indicating potential small‐study effects and the likelihood of publication bias. The clustering of studies within areas denoting statistical significance further reinforces concerns that are non‐significant. To explore this further, we conducted a trim‐and‐fill analysis (Figure S1). The method imputed one potentially missing study on the right side of the funnel plot, suggesting that the overall pooled estimate might be subject to modest inflation due to the selective publication of studies reporting larger treatment effects.

### 3.9 Certainty of Evidence

The certainty of the evidence was rated very low due to serious concerns in four domains: risk of bias (6 of 8 studies had some concerns or were non‐randomized), inconsistency (*I* ^2^ = 85.4%), imprecision (wide CI and prediction interval from −4.20 to 0.82), and indirectness (variation in outcome measures, study designs, and populations) (Table 4).

**Table 4:** Summary of findings table according to the GRADE approach.

**Efficacy of self‐assembling peptide in reducing white spot lesions**				
**Review question**: Is SAP P11‐4 more effective than fluoride or no treatment in reducing white spot lesions (WSLs)?				
**Population**: Orthodontic patients or extracted teeth with WSLs				
**Settings**: Egypt, Germany, Turkey				
**Intervention**: Self‐assembling peptide (SAP P11‐4)				
**Comparison**: Fluoride or no treatment				

**Outcomes**	**Number of participants (studies)**	**Certainty of the evidence (GRADE)**	**Difference with experimental group**	**What happens with experimental treatment**
WSL quantification	395 (8 studies)	⊕◯◯◯ Very low^a^^,^ b ^,^ c ^,^ d	SMD 1.68 SD lower (2.71 lower to 0.67 lower)	SAP P11‐4 may reduce WSLs more than fluoride or control, but the evidence is very uncertain

Abbreviations: CI, confidence interval; NRS, non‐randomized studies; SMD, standardized mean difference.

^a^ Risk of bias: Most included studies were at some concern level (6 out of 8), and several were non‐randomized or had unclear blinding.

^b^ Inconsistency: High heterogeneity (*I* ^2^ = 85.4%) with wide variability in outcomes and methods.

^c^ Imprecision: Confidence interval ranges from a moderate to large effect to a small effect, and prediction interval (−4.20 to 0.82) includes null and opposite effects.

^d^ Indirectness: Variability in outcome measures (fluorescence loss, WSL score, microhardness, etc.), designs (in‐vitro and clinical), and patient populations.

## 4 Discussion

This is the first meta‐analysis to include eight clinical studies involving a total of 395 participants, assessing the efficacy of SAP compared to fluoride or control groups. SAP demonstrated a statistically significant and large effect in reducing WSLs, with an SMD of −1.68 (95% CI: −2.71 to −0.67; *p* = 0.005). This substantial effect can be attributed to the unique biomimetic action of SAPs, which infiltrate subsurface enamel lesions and form a three‐dimensional scaffold that mimics natural enamel matrix (Alkilzy et al. 2023). This structure promotes deep remineralization by guiding calcium and phosphate deposition. Unlike fluoride, which primarily acts at the surface and may block deeper repair, SAP enables uniform regeneration throughout the lesion (Kamal et al. 2018). Its lesion‐guided, minimally invasive mechanism aligns with modern caries management and explains the consistently favorable outcomes and large effect size observed in this meta‐analysis.

Strategies for the prevention and management of WSLs encompass a wide range of approaches, including fluoride‐based products such as varnishes, mouthwashes, toothpastes, and fluoride‐releasing adhesives (Shah et al. 2018; Stecksén‐Blicks et al. 2007; Sonesson et al. 2014, 2020; Wang et al. 2023; Sardana et al. 2023); bioactive glasses including calcium sodium phosphosilicate (NovaMin) (Tiwari and Jain 2023); antibacterial agents like chlorhexidine, ozone, and probiotics (Tiwari and Jain 2023; Grocholewicz et al. 2022); bonding‐related modifications including resin‐modified glass ionomer, self‐etching primers, and resin infiltration techniques (Kashash et al. 2024; Horan and Al‐Khateeb 2023); as well as nanotechnology‐based agents such as nanosilver and titanium dioxide nanoparticles (Al Tuma and Yassir 2023).

These agents act through multiple mechanisms to prevent WSL formation. Fluoride facilitates enamel remineralization, suppresses bacterial activity, and increases resistance to acid challenges. CPP‐ACP, in contrast, provides a sustained source of bioavailable calcium and phosphate ions at the tooth surface, thereby enhancing the remineralization process (Esenlik et al. 2016). NovaMin enhances enamel remineralization by releasing calcium and phosphate ions that attach to the tooth surface and promote hydroxycarbonate apatite formation (Mollabashi et al. 2022). Antibacterial agents suppress bacterial growth and disrupt dental biofilms, creating favorable conditions for enamel remineralization from salivary minerals. Due to their small particle size and high surface area, nano‐agents interact more effectively with bacterial cells, enabling penetration and bacterial destruction. As a result, these agents exhibit strong anti‐plaque and anti‐cariogenic effects (Al Tuma and Yassir 2023; Ali, Ismail et al. 2022). A new treatment modality called SAP for WSLs, which promotes biomimetic mineralization, has been introduced. SAP works by infiltrating the subsurface lesion, which self‐assembles into a nanofiber matrix (Alkilzy et al. 2018). This structure attracts calcium ions from saliva and templates hydroxyapatite formation, promoting natural enamel remineralization. By enhancing the inherent repair mechanisms of enamel, SAP offers a non‐invasive treatment (Kind et al. 2017).

In this review, all included studies assessed white spot lesions (DLs), but outcomes were measured using different scales, including fluorescence loss, surface microhardness, color change, and WSL severity scores. To allow meaningful synthesis, we used SMDs to standardize the effect sizes across studies. Heterogeneity was quantified using *I* ^2^ and *τ* ^2^, reflecting variability in effects, and GRADE assessment was performed to evaluate the certainty of evidence. Our active intervention was SAP, with all other groups serving as controls, and sensitivity analyses were conducted to explore differences across subgroups such as control type, study design, ethnicity, outcome measures, and risk of bias. These analyses demonstrated that the overall results were robust despite variations in control interventions and outcome measures.

Statistically, the large effect size (SMD = −1.68; 95% CI: −2.71 to −0.67) reflects consistent superiority of SAP over control across multiple trials with relatively low within‐study variability. However, assessment of publication bias revealed funnel plot asymmetry, and Egger's test indicated potential small‐study effects. To address this, the trim‐and‐fill method was applied, imputing one potentially missing study. Even after adjustment, the effect remained statistically significant, suggesting that while the magnitude may be slightly overestimated, the clinical benefit of SAP in reducing WSLs remains robust and biologically justified. Furthermore, to reduce the risk of spurious findings due to between‐study heterogeneity or small sample sizes, we applied Hedges' *g* (Taylor and Alanazi 2023) for effect size estimation and used the Hartung–Knapp adjustment (Röver et al. 2015) for more conservative confidence intervals. These corrections confirmed the statistical significance of the findings and strengthen the reliability of the observed treatment effect.

Our results are in agreement with the recent two systematic reviews and meta‐analyses on non‐orthodontic patients by Wierichs et al. (2021), which synthesized seven RCTs involving 294 patients. Their review reported significant improvement in SAP‐treated lesions based on laser fluorescence (SMD = 0.87; 95% CI: 0.34–1.39) and visual analog scale scores (MD = 35.38; 95% CI: 27.64–43.13). Similarly, another systematic review by Rathore et al. (2023) assessed the effectiveness of SAP with or without fluoride agents (FA) in the management of WSLs/incipient carious lesions. Their findings found benefit, particularly when SAP was combined with fluoride (SAP + FA vs. FA: MD = −11.52; 95% CI: −14.43 to −8.61; *p* < 0.001).

Heterogeneity was assessed through forest plots, the *I* ^2^ statistic (85.4%), and *τ*², which was estimated using the restricted maximum likelihood (REML) method. Substantial heterogeneity was found in our meta‐analysis assessing the efficacy of SAP for managing WSLs in orthodontic patients (*I* ^2^ = 85.4%; 95% CI: 73.2%–92.1%), with considerable between‐study variance (*τ* ^2^ = 0.97; 95% CI: 0.31–6.98) and a wide prediction interval (−4.20 to 0.82). This heterogeneity can be attributed to multiple sources, including differences in ethnicity, study design (RCTs, non‐randomized trials, in‐vitro studies), dispensing methods and concentrations of SAP or comparators (fluoride or no treatment), method of outcome measures (WSL scoring indices, fluorescence loss, microhardness), patients' age, and follow‐up duration.

Heterogeneity was further explored through sensitivity analyses. Subgroup comparisons revealed a significantly stronger effect in RCTs (SMD = −2.18) than in non‐randomized studies (SMD = −0.68), with a significant difference across study designs (*p* = 0.011). Sensitivity analysis was also conducted by excluding one in‐vitro study from the pooled effect, and the results remained robust: overall pooled effect (−1.68 [−2.70; −0.67]) versus without the in vitro study (−1.76[−2.9; −0.53). No substantial differences were found by ethnicity (*p* = 0.74), comparator type (fluoride vs. no treatment; *p* = 0.79), outcome measure (*p* = 0.21), or risk of bias (*p* = 0.74).

The current evidence for the effect was rated as very low according to the GRADE framework. This was due to several limitations: most studies had a risk of bias, with six out of eight rated as having “some concern,” and several were non‐randomized or lacked blinding. There was substantial inconsistency across outcomes and study designs, contributing to heterogeneity. Imprecision was also evident, as indicated by wide confidence and prediction intervals, reflecting uncertainty about the true effect size. Furthermore, indirectness was present due to variability in outcome measures and study populations, such as in vitro versus clinical settings, reducing the generalizability of the findings.

The prediction interval shows the range in which the effect of future studies is likely to fall, considering differences between studies. In our analysis, the wide interval (–4.20 to 0.82) reflects substantial variation among studies, meaning that while the overall effect favors one group, individual future studies could find smaller, larger, or even opposite effects. This indicates that the observed benefit may not be consistent across all populations or clinical settings, and results should be applied cautiously.

The clinical implication of this study is that SAP represents a promising adjunct or alternative to traditional preventive strategies for orthodontic WSLs. Its minimally invasive, lesion‐targeted mechanism allows subsurface remineralization, potentially improving esthetic outcomes and reducing the need for restorative interventions post‐orthodontic treatment. While fluoride and bioactive agents remain standard care, SAP may be particularly useful in high‐risk patients with early lesions or poor compliance, or as part of combination therapy with fluoride. Future high‐quality, multicenter RCTs with standardized outcome measures are needed to confirm optimal protocols, dosing, and long‐term efficacy in routine orthodontic practice.

The issue with the unit of analysis should be considered when interpreting the results. To address potential unit‐of‐analysis concerns, we analyzed the reported outcomes as presented in the original studies. For studies reporting multiple teeth per participant (e.g., split‐mouth RCTs or in vitro studies), we documented the study design and considered the inherent clustering when interpreting effect estimates and precision. Although formal adjustment for clustering (e.g., using multilevel models) was not possible due to the absence of individual‐level data, this limitation should be acknowledged, as it may lead to an overestimation of precision. The discrepancy between the number of teeth and the number of patients as the unit of analysis primarily arose from the inclusion of both human clinical studies and in vitro studies. To address this issue, we conducted a subgroup analysis according to study type, allowing the effects of human and in vitro studies to be evaluated separately (Table 3).

## 5 Strengths and Limitations

This is the first meta‐analysis to evaluate the clinical effectiveness of SAPs for WSLs in orthodontic patients. Strengths include a priori registration, transparent reporting, inclusion of only human studies, and the application of robust statistical methods. Limitations include the small number of included studies, inclusion of in vitro studies, and high statistical heterogeneity. Additionally, non‐randomized studies reducing the overall certainty of evidence.

## 6 Future Prospects

Future studies should explore the optimal concentration and application frequency of SAPs for orthodontic patients, evaluate the long‐term stability of remineralized enamel, and assess patient‐centered outcomes such as esthetic satisfaction and quality of life. Additionally, future studies should investigate combination therapies integrating SAP with fluoride, CPP‐ACP, or bioactive glass to determine potential synergistic effects. The development of chairside delivery systems or slow‐release formulations should also be considered to enhance clinical practicality and patient adherence. Finally, future studies should be conducted as multicenter, high‐quality RCTs with standardized outcome measures, longer follow‐up periods, and diverse populations to establish robust and generalizable evidence for routine clinical use.

## 7 Conclusion

Very low‐level evidence suggests that SAPs can reduce the severity of WSLs associated with fixed orthodontic treatment more effectively than topical fluoride or no treatment. However, further RCTs with larger sample sizes and longer follow‐up periods are needed to establish stronger evidence.

## Author Contributions

**Sheema Shakir:** data curation (equal), risk of bias (equal), investigation (equal), writing – review and editing (equal). **Farhana Umer:** conceptualization (equal), data extraction (equal), writing – review and editing (equal). **Qasim Khalid:** conceptualization (equal), investigation (equal), methodology (equal), writing – review and editing (equal). **Khurram Shahzad:** data curation (equal), risk of bias (equal), investigation (equal), writing – review and editing (equal). **Umar Hussain:** conceptualization (equal), investigation (equal), methodology (equal), project administration (equal), writing – original draft (equal), software (equal), formal analysis (equal). **Samrina Mohammad:** conceptualization (equal), investigation (equal), methodology (equal), writing – review and editing (equal). **Hamna Omair:** data curation (equal), risk of bias (equal), investigation (equal), writing – review and editing (equal). **Alessandra Campobasso:** conceptualization (equal), investigation (equal), methodology (equal), writing – review and editing (equal). **Waqas Naseem:** conceptualization (equal), investigation (equal), methodology (equal), writing – review and editing (equal). **Junad Khan:** supervision (equal), investigation (equal), methodology (equal), project administration (equal), writing – original draft (equal), final review (equal).

## Funding

The authors received no specific funding for this work.

## Ethics Statement

This study is a systematic review and does not involve human participants, patient data, or animal subjects.

## Consent

As this meta‐analysis is based solely on previously published data and does not involve human participants, no consent was required.

## Conflicts of Interest

The authors whose names are listed in title page certify that they have no affiliations with or involvement in any organization or entity with any financial interest (such as honoraria; educational grants; participation in speakers' bureaus; membership, employment, consultancies, stock ownership, or other equity interest; and expert testimony or patent‐licensing arrangements), or non‐financial interest (such as personal or professional relationships, affiliations, knowledge or beliefs) in the subject matter or materials discussed in this manuscript.

## Supporting information


**Figure S1:** Trim‐and‐Fill Funnel Plot of WSL Quantification. **Figure S2:** Forest plot showing subgroup analysis of the efficacy of self‐assembling peptide by type of comparator. **Table S1:** Searches from five databases. **Table S2:** List of included/excluded studies.

## Data Availability

All data is openly available through Zenodo https://zenodo.org/records/16579132.
