# Supplementary material for: Effect of Self‐Assembling Peptide on White Spot Lesions in Orthodontic Patients: A Systematic Review and Meta‐Analysis
Source: Clin Exp Dent Res. 2026 Feb 26;12(2):e70321. doi: 10.1002/cre2.70321 (PMC12944740; doi:10.1002/cre2.70321)
Supplement: Supplementary file 1 — Figure S1: Trim‐and‐Fill Funnel Plot of WSL Quantification. Figure S2: Forest plot showing subgroup analysis of the efficacy of self‐assembling peptide by type of comparator. Table S1: Searches from five databases. Table S2: List of included/excluded studies. [file CRE2-12-e70321-s001.docx]

**Supplementary file**

**Effect of Self-Assembling Peptide on White Spot Lesions in Orthodontic Patients: A Systematic Review and Meta-Analysis**

- Outcome variables such as mineral content were pooled together with WSL scores, as all reflect quantitative measures of white spot lesions. During analysis, certain variables were reversed—for example, mineral content and micro-hardness values for the control group were swapped with those of the experimental group—because an increase in mineral content indicates treatment efficacy, whereas a decrease in WSL scores signifies improvement.
- In the study by Riad (2020), the corresponding author was contacted, and she kindly provided the requested data.

Table S1. Searches from five databases (last date of search: 16/5/2025)

| Database | Keywords | Filter Applied | Hits |
| --- | --- | --- | --- |
| PubMed | ("Peptide*" OR "self-assembling" OR "self assembly" OR "SAP" OR "SAPs" OR "P11-4" OR "biomimetic" OR "remineralizing" OR "peptide hydrogel*" OR "amphiphilic peptide*" OR "enamel matrix derivative*" OR "EMD") AND ("Tooth Demineralization" OR "Dental Caries" OR "white spot" OR "WSL" OR "enamel demineral*" OR "decalcification" OR "early caries" OR "incipient caries" OR "carious lesion*" OR "enamel lesion*") AND ("Orthodontics" OR "Orthodontic Appliances" OR "orthodontic" OR "fixed appliance*" OR "braces" OR "bracket*" OR "orthodontic treatment" OR "orthodontic patient*") | All Fields | 203 |
| Scopus | Same as above | Article title, abstract, keywords | 178 |
| WOS (Web of Science) | Same as above | Topic (title, abstract, keyword plus, author keywords) | 133 |
| Cochrane Library | Same as above | Title, Abstract, Keywords | 1 review + 134 trials |
| LILACS | Same as above | Title, Abstract, Subject | 9 |


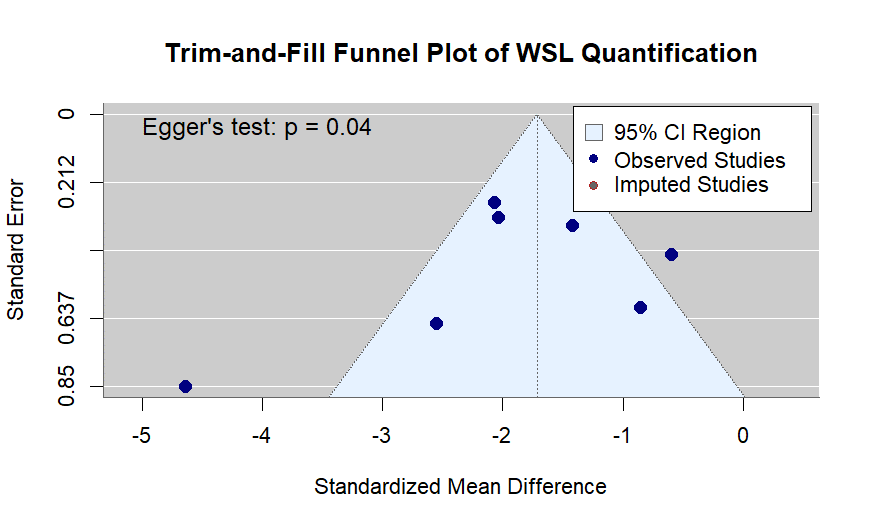


Fig S1. Trim-and-Fill Funnel Plot of WSL Quantification


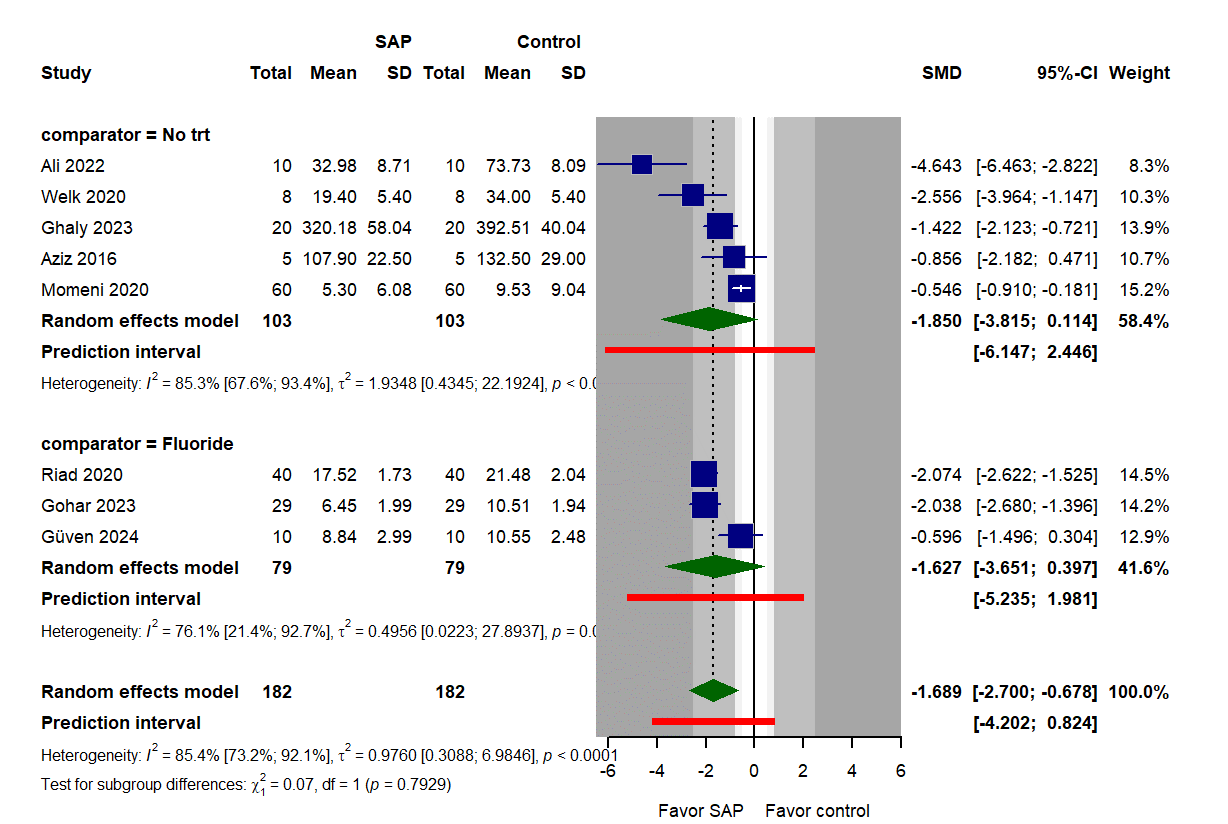


Fig S2. Forest plot showing subgroup analysis of the efficacy of self-assembling peptide by type of comparator

Table S2. List of included/excluded studies

| SNo | Author(s) | Title | by | reason for exclusion/ inclusion | Year |
| --- | --- | --- | --- | --- | --- |
| 1 | Ogihara, | Efficacy of forced eruption/enamel matrix derivative with freeze-dried bone allograft or with demineralized freeze-dried bone allograft in infrabony defects: a randomized trial.2015 | title/abstract | irrelevant | 2015 |
| 2 | Jablonski-Mo | Impact of self-assembling peptides in remineralisation of artificial early enamel lesions adjacent to orthodontic brackets.2020 | fulltext | included | 2020 |
| 3 | Welk, A; Rat | Effect of self-assembling peptide P11-4 on orthodontic treatment-induced carious lesions.2020 | fulltext | included | 2020 |
| 4 | NCT04992481, | Fluoride Varnish, Ozone, Octenidin and WSLs in Orthodontic Patients.2021 | title/abstract | registry | 2021 |
| 5 | Benson, PE; | An in situ caries model to study demineralisation during fixed orthodontics.1999 | title/abstract | irrelevant | 1999 |
| 6 | Garry, AP; F | A randomised controlled trial to investigate the remineralising potential of Tooth Mousseâ„¢ in orthodontic patients.2017 | fulltext | ineligible intervention | 2017 |
| 7 | NCT03966378, | Reminerlization Potential of GSE Gel versusCPP-ACP in Carious White Spot Lesions in Post Orthodontic Patients.2019 | title/abstract | registry | 2019 |
| 8 | Flynn, LN; J | The efficacy of fluoride varnish vs a filled resin sealant for preventing white spot lesions during orthodontic treatment.2022 | title/abstract | irrelevant | 2022 |
| 9 | Mollabashi, | Evaluation of Nano TiO2 Modified Orthodontic Composite Effects on S. mutans Population and Enamel Demineralization in Fixed Orthodontic Patients; a Split Mouth Randomized Controlled Clinical Trial.2023 | duplicate | duplicate | 2023 |
| 10 | Robertson, M | MI Paste Plus to prevent demineralization in orthodontic patients: a prospective randomized controlled trial.2011 | fulltext | ineligible intervention | 2011 |
| 11 | NCT05733676, | Comparison of MI Paste Plus and Resin Infiltration in White Spot Lesions Following Orthodontic Treatment.2023 | title/abstract | registry | 2023 |
| 12 | ACTRN1260500 | Remineralisation of decalcified tooth enamel consequent to orthodontic treatment.2005 | title/abstract | registry | 2005 |
| 13 | NCT01059058, | Study of Treatments Used for White Spot Lesions After Orthodontic Treatment.2010 | title/abstract | registry | 2010 |
| 14 | Mensinkai, P | In situ remineralization of white-spot enamel lesions by 500 and 1,100 ppm F dentifrices.2012 | title/abstract | irrelevant | 2012 |
| 15 | NCT01329731, | Remineralisation of White Spot Lesions by ElmexÂ® gelÃ©e in Post-orthodontic Patients.2011 | title/abstract | registry | 2011 |
| 16 | Beerens, MW; | Effects of casein phosphopeptide amorphous calcium fluoride phosphate paste on white spot lesions and dental plaque after orthodontic treatment: a 3-month follow-up.2010 | title/abstract | ineligible intervention | 2010 |
| 17 | Restrepo, M; | Control of White Spot Lesions with Use of Fluoride Varnish or Chlorhexidine Gel During Orthodontic Treatment A Randomized Clinical Trial.2016 | title/abstract | ineligible intervention | 2016 |
| 18 | Badiee, M; J | Comparison of the effects of toothpastes containing nanohydroxyapatite and fluoride on white spot lesions in orthodontic patients: a randomized clinical trial.2020 | duplicate | duplicate | 2020 |
| 19 | Badiee, M; J | Comparison of the effects of toothpastes containing nanohydroxyapatite and fluoride on white spot lesions in orthodontic patients: a randomized clinical trial.2020 | duplicate | duplicate | 2020 |
| 20 | Silva, VM; M | Prevention of non-cavitated lesions with fluoride and xylitol varnishes during orthodontic treatment: a randomized clinical trial.2021 | title/abstract | ineligible intervention | 2021 |
| 21 | Mathews, MS; | In situ remineralisation of eroded enamel lesions by NaF rinses.2012 | title/abstract | irrelevant | 2012 |
| 22 | Pavethynath, | Evaluation of Prevention of Initial Enamel Lesions around Orthodontic Brackets by Using Different Remineralizing Agents: an Original Research.2024 | fulltext | ineligible intervention | 2024 |
| 23 | Beerens, MW; | Long-term remineralizing effect of MI Paste Plus on regression of early caries after orthodontic fixed appliance treatment: a 12-month follow-up randomized controlled trial.2018 | fulltext | ineligible intervention | 2018 |
| 24 | SkÃ¶ld-Larss | Effect of fluoridated milk on enamel demineralization adjacent to fixed orthodontic appliances.2013 | title/abstract | ineligible intervention | 2013 |
| 25 | Terakulvanic | Fluoridated milk enhances the mineral density of artificial proximal carious lesions in situ.2022 | title/abstract | irrelevant | 2022 |
| 26 | CTRI/2022/05 | Comparative evaluation of effect of varnishes on white spot lesions in orthodontic patients: an in Vivo in Vitro randomized controlled trial.2022 | title/abstract | registry | 2022 |
| 27 | NCT04827966, | Remineralization Agents for the Treatment of White Spot Lesions.2021 | title/abstract | registry | 2021 |
| 28 | Willmot, DR | White lesions after orthodontic treatment: does low fluoride make a difference?.2004 | title/abstract | irrelevant | 2004 |
| 29 | CTRI/2020/12 | Remineralization efficacy of CPP-ACP with Fluoride versus Sodium Fluoride mouthrinse on white spot lesions among orthodontic patients.2020 | title/abstract | registry | 2020 |
| 30 | Aljehani, A; | Longitudinal quantification of incipient carious lesions in postorthodontic patients using a fluorescence method.2006 | title/abstract | irrelevant | 2006 |
| 31 | NCT03823612, | Reminerlization Potential of Innovative Biomimetic Material in Caries White Spot Lesions.2019 | title/abstract | registry | 2019 |
| 32 | Adeyemi, AA; | Demineralisation following orthodontic treatment: recruitment to an RCT.2007 | title/abstract | irrelevant | 2007 |
| 33 | CTRI/2022/07 | A new Innovative Oral Bioadhesive Formulation Incorporated With Nano Hydroxyapatite and Acorus Calamus Rhizome Extract To Enhance Remineralization of White Spot Lesions during braces Treatment Ã¢?? A Randomized Controlled Trial.2022 | title/abstract | registry | 2022 |
| 34 | Behnan, SM; | In-vitro evaluation of various treatments to prevent demineralization next to orthodontic brackets.2010 | title/abstract | ineligible intervention | 2010 |
| 35 | Knaup, T; Ko | Effect of the caries-protective self-assembling peptide P11-4 on shear bond strength of metal brackets.2021 | fulltext | ineligible outcome | 2021 |
| 36 | Gray, A; Fer | The use of low-tack chewing gum for individuals wearing orthodontic appliances.1996 | title/abstract | irrelevant | 1996 |
| 37 | NCT06797882, | Preventive Effects of Silver Diamine Fluoride on Early Enamel Caries.2025 | title/abstract | registry | 2025 |
| 38 | Du, M; Cheng | Randomized controlled trial on fluoride varnish application for treatment of white spot lesion after fixed orthodontic treatment.2012 | title/abstract | irrelevant | 2012 |
| 39 | NCT01344473, | A Trial of Tooth Mousse to Remineralise Post-orthodontic Treatment White Spot Lesions.2011 | title/abstract | registry | 2011 |
| 40 | O'Reilly, MM | Demineralization and remineralization around orthodontic appliances: an in vivo study.1987 | title/abstract | irrelevant | 1987 |
| 41 | NCT04342858, | Prospective Randomized Controlled Trial for Prevention of Demineralization During Fixed Orthodontic Treatment.2020 | title/abstract | registry | 2020 |
| 42 | Bansal, K; G | In vivo remineralization of artificial enamel carious lesions using a mineral-enriched mouthrinse and a fluoride dentifrice: a polarized light microscopic comparative evaluation.2010 | title/abstract | ineligible intervention | 2010 |
| 43 |  | Sodium fluoride mouthrinse used twice daily increased incipient caries lesion remineralization in an in situ model.2014 | title/abstract | irrelevant | 2014 |
| 44 | Songsiriprad | Sodium fluoride mouthrinse used twice daily increased incipient caries lesion remineralization in an in situ model.2014 | title/abstract | irrelevant | 2014 |
| 45 | NCT05940701, | The Effectiveness of Biomimetic Materials in Preventing Enamel Demineralization During Fixed Orthodontic Treatment.2023 | title/abstract | registry | 2023 |
| 46 | Adeyemi, AA; | Measuring enamel-remineralisation during the retention phase of orthodontic treatment.2008 | title/abstract | irrelevant | 2008 |
| 47 | Rechmann, P; | MI Varnish and MI Paste Plus in a caries prevention and remineralization study: a randomized controlled trial.2018 | title/abstract | irrelevant | 2018 |
| 48 | KnÃ¶sel, M; | Efficacy of different strategies in protecting enamel against demineralization during fixed orthodontic treatment.2012 | title/abstract | irrelevant | 2012 |
| 49 | Wierichs, RJ | Short-term efficacy of caries resin infiltration during treatment with orthodontic fixed appliances. A randomized controlled trial.2023 | title/abstract | irrelevant | 2023 |
| 50 | Bhongsatiern | Adjunctive use of fluoride rinsing and brush-on gel increased incipient caries-like lesion remineralization compared with fluoride toothpaste alone in situ.2019 | title/abstract | irrelevant | 2019 |
| 51 | NCT04017884, | Evaluation of Remin Pro Forte Versus Remin Pro on Treatment of White Spot Lesions Post Orthodontic Treatment.2019 | title/abstract | registry | 2019 |
| 52 | Kau, CH; Wan | Effect of fluoride dentifrices on white spot lesions during orthodontic treatment: a randomized trial.2019 | title/abstract | ineligible intervention | 2019 |
| 53 | CTRI/2024/02 | Testing the efficacy of remineralising toothpaste containing calcium sucrose phospate, casein phospopeptide amorphous calcium phosphate fluoride and nanohydroxyapatite in the prevention of white spot lesions in people with metal braces.2024 | title/abstract | registry | 2024 |
| 54 | Akin, M; Bas | Can white spot lesions be treated effectively?.2012 | title/abstract | irrelevant | 2012 |
| 55 | Babanouri, N | Effect of high concentration nano-hydroxyapatite serum on shear bond strength of metal brackets following three different enamel surface preparation methods: an in vitro study.2021 | title/abstract | irrelevant | 2021 |
| 56 | Wulc, D; God | Efficacy of fluoride varnish in management of orthodontic white spots.2015 | title/abstract | irrelevant | 2015 |
| 57 | Bailey, DL; | Regression of post-orthodontic lesions by a remineralizing cream.2009 | title/abstract | irrelevant | 2009 |
| 58 | Moraes, SM; | Effectiveness of Fluoride Varnishes for White Spot Lesion Prevention and Remineralization during Orthodontic Treatment: a Randomized Controlled Trial.2024 | title/abstract | irrelevant | 2024 |
| 59 | Verma, P; Mu | Bionic effects of nano hydroxyapatite dentifrice on demineralised surface of enamel post orthodontic debonding: in-vivo split mouth study.2021 | title/abstract | irrelevant | 2021 |
| 60 | CTRI/2018/11 | Comparing two different products i.e. fluoride varnish and resin infiltration for treatment of white spot lesion after orthodontic treatment.2018 | title/abstract | registry | 2018 |
| 61 | Sain, S; Heg | Remineralization of Enamel Using Topical Agents among Patients with Orthodontic Brackets: in Vivo and In Vitro Randomized Control Trial.2024 | duplicate | duplicate | 2024 |
| 62 | CTRI/2020/03 | Efficiency of remineralising agents on white spots lesions during braces treatment- a controlled clinical trial.2020 | title/abstract | registry | 2020 |
| 63 | NCT06749977, | P11-4 and Fluoride Varnish for Remineralization of Post-orthodontic Lesions.2024 | title/abstract | registry | 2024 |
| 64 | Ogaard, B; R | Orthodontic appliances and enamel demineralization. Part 2. Prevention and treatment of lesions.1988 | title/abstract | ineligible intervention | 1988 |
| 65 | Johal, A; Ke | The effectiveness of Bio-Min toothpaste in the management of white spot lesions: a randomised control trial.2024 | title/abstract | ineligible intervention | 2024 |
| 66 | NCT05948228, | Prevention of White Spot Lesions Using Bioactive Gel.2023 | title/abstract | registry | 2023 |
| 67 | NCT03360266, | Evaluation of Remineralizing Efficacy of Combination Varnishes on White Spot Lesions in ECC Children.2017 | title/abstract | registry | 2017 |
| 68 | NCT04788550, | The Effect of Different Remineralizing Agents on White Spot Lesions and Dental Plaque During Orthodontic Retention.2021 | title/abstract | registry | 2021 |
| 69 | Gohar, RAAEG | Evaluation of the remineralizing effect of biomimetic self-assembling peptides in post-orthodontic white spot lesions compared to fluoride-based delivery systems: randomized controlled trial.2023 | duplicate | duplicate | 2023 |
| 70 | NCT02424097, | MI Varnish and MI Paste Plus in a Caries Prevention and Remineralization Study.2015 | title/abstract | registry | 2015 |
| 71 | RBR-104n8kqh | Prevention and remineralization of recent dental caries injuries in Orthodontics: comparison of the efficacy between Sodium Fluoride Varnish and Titanium Tetrafluoride Varnish (TiF4).2021 | title/abstract | irrelevant | 2021 |
| 72 | NCT03976583, | Remineralization Potential of Pearl Powder Compared to CPP-ACP on Enamel White Spot Lesions.2019 | title/abstract | registry | 2019 |
| 73 | Mollabashi, | DIAGNOdent pen quantification of the synergy of NovaMinÂ® in fluoride toothpaste to remineralize white spot lesions in patients with fixed orthodontic appliances: a double-blind, randomized, controlled clinical trial.2022 | title/abstract | irrelevant | 2022 |
| 74 | IRCT20190827 | Effect of flouride mouthwash and tooth-mousse paste on fixed orthodontic appliances.2019 | title/abstract | registry | 2019 |
| 75 | NCT02913885, | Effect of Curodont Repair on Color Change of White Spot Lesions.2016 | title/abstract | registry | 2016 |
| 76 | He, T; Li, X | Comparative assessment of fluoride varnish and fluoride film for remineralization of postorthodontic white spot lesions in adolescents and adults over a 6-month period: a single-center, randomized controlled clinical trial.2016 | title/abstract | irrelevant | 2016 |
| 77 | Jablonski-Mo | Randomised in situ clinical trial investigating self-assembling peptide matrix P11-4 in the prevention of artificial caries lesions.2019 | duplicate | duplicate | 2019 |
| 78 | Trairatvorak | Effect of glass ionomer cement and fluoride varnish on the remineralization of artificial proximal caries in situ.2009 | title/abstract | ineligible intervention | 2009 |
| 79 | NCT06823310, | Antibiofilm Activity of Chitosan Nanoparticles Incorporated Into Clear Aligners.2025 | title/abstract | registry | 2025 |
| 80 | NCT06178042, | Effectiveness of MI Paste Plus and Remin Pro on Remineralization of Post-Orthodontic White Spot Lesions.2023 | title/abstract | registry | 2023 |
| 81 | Baeshen, HA; | Effect of fluoridated chewing sticks (Miswaks) on white spot lesions in postorthodontic patients.2011 | duplicate | duplicate | 2011 |
| 82 | El Sayed, M; | Efficacy of fluoride varnish containing casein phosphopeptide-amorphous calcium phosphate application and diode laser irradiation on white spot lesions remineralization: an in vitro study.2025 | title/abstract | irrelevant | 2025 |
| 83 | Uysal, T; Am | Amorphous calcium phosphate-containing orthodontic composites. Do they prevent demineralisation around orthodontic brackets?.2010 | title/abstract | irrelevant | 2010 |
| 84 | Zhang, C; Xu | The Application of Resin Infiltration Combined with Nano-Hydroxyapatite in the Treatment of Post-Orthodontic White Spot Lesions.2024 | title/abstract | irrelevant | 2024 |
| 85 | Restrepo, M; | Control of white spot lesion adjacent to orthodontic bracket with use of fluoride varnish or chlorhexidine gel.2015 | title/abstract | irrelevant | 2015 |
| 86 | Mayne, RJ; C | In-vitro study of the effect of casein phosphopeptide amorphous calcium fluoride phosphate on iatrogenic damage to enamel during orthodontic adhesive removal.2011 | title/abstract | irrelevant | 2011 |
| 87 | Visel, D; JÃ | Demineralization adjacent to orthodontic brackets after application of conventional and self-etching primer systems.2014 | title/abstract | irrelevant | 2014 |
| 88 | ISRCTN144798 | How effective are toothpastes in improving tooth discoloration marks after brace treatment?.2020 | title/abstract | registry | 2020 |
| 89 | KarabekiroÄŸ | Effectiveness of different remineralization agents for treatment of white spot lesions: a randomized controlled trial.2015 | fulltext | ineligible intervention | 2015 |
| 90 | NCT01925924, | RMGI v Composite for Orthodontic Bonding.2013 | title/abstract | registry | 2013 |
| 91 | BrÃ¶chner, A | Treatment of post-orthodontic white spot lesions with casein phosphopeptide-stabilised amorphous calcium phosphate.2011 | title/abstract | irrelevant | 2011 |
| 92 | Baysal, A; U | Evaluation of enamel demineralization in adolescents after rapid maxillary expansion using the quantitative light-induced fluorescence method: a single-center, randomized controlled clinical trial.2016 | title/abstract | irrelevant | 2016 |
| 93 | Smyth, RSD; | Preventing white spot lesions with fluoride pastes.2019 | title/abstract | irrelevant | 2019 |
| 94 | Gokce, G; Sa | Effects of toothpastes on white spot lesions around orthodontic brackets using quantitative light-induced fluorescence (QLF) : an in vitro study.2017 | title/abstract | irrelevant | 2017 |
| 95 | Nantanee, R; | Silver diamine fluoride and glass ionomer differentially remineralize early caries lesions, in situ.2016 | title/abstract | irrelevant | 2016 |
| 96 | CTRI/2012/04 | Dental caries prevention in fixed orthodontic therapy.2012 | title/abstract | registry | 2012 |
| 97 | Taha, AA; Fl | Enamel Remineralization with Novel Bioactive Glass Air Abrasion.2018 | title/abstract | irrelevant | 2018 |
| 98 | ChiCTR250009 | Study on the remineralization effect of enamel demineralization based on a mineralized treatment system of amorphous calcium phosphate funtionally modified with column aromatics.2025 | title/abstract | irrelevant | 2025 |
| 99 | Riad, MF; Ra | Comparative study using biomimetic remineralization versus fluoride varnish in management of white spot lesion in post orthodontic treated patient: split mouth randomized clinical trial.2020 | fulltext | included | 2020 |
| 100 | Lena Sezici, | Comparative evaluation of fluoride varnishes, self-assembling peptide-based remineralization agent, and enamel matrix protein derivative on artificial enamel remineralization in vitro.2021 | title/abstract | irrelevant | 2021 |
| 101 | Andersson, A | Effect of a dental cream containing amorphous cream phosphate complexes on white spot lesion regression assessed by laser fluorescence.2007 | title/abstract | ineligible intervention | 2007 |
| 102 | Wan Hassan, | A 12-months randomized clinical trial comparing fluoride-based remineralising protocols on post-orthodontic initial caries lesions.2025 | duplicate | duplicate | 2025 |
| 103 | Singh, S; Si | Effects of various remineralizing agents on the outcome of post-orthodontic white spot lesions (WSLs): a clinical trial.2016 | title/abstract | ineligible intervention | 2016 |
| 104 | KarabekiroÄŸ | Treatment of post-orthodontic white spot lesions with CPP-ACP paste: a three year follow up study.2017 | title/abstract | irrelevant | 2017 |
| 105 | CTRI/2020/01 | A clinical trial to compare the effectiveness of two methods of remineralisation using CPP-ACP varnish on white spots on teeth.2020 | title/abstract | registry | 2020 |
| 106 | Aboulnaga, M | Evaluation of Remineralization Potential of Remin Pro Forte vs Remin Pro on White Spot Lesions: a Randomized Clinical Trial.2022 | duplicate | duplicate | 2022 |
| 107 | Bock, NC; Se | Changes in white spot lesions following post-orthodontic weekly application of 1.25 per cent fluoride gel over 6 months-a randomized placebo-controlled clinical trial. Part II: clinical data evaluation.2017 | title/abstract | ineligible intervention | 2017 |
| 108 | Bock, NC; Se | Changes in white spot lesions following post-orthodontic weekly application of 1.25 per cent fluoride gel over 6 months-a randomized placebo-controlled clinical trial. Part I: photographic data evaluation.2017 | title/abstract | ineligible intervention | 2017 |
| 109 | NCT04538963, | Arrest of Proximal Caries Using Orthodontic Bands and Glass Ionomer Cement.2020 | title/abstract | registry | 2020 |
| 110 | Rangappa, KG | Remineralization Potential of a Combination of Chitosan with Nanohydroxyapatite and a Self-assembling Peptide with Nanohydroxyapatite: an In Vitro Study.2022 | duplicate | duplicate | 2022 |
| 111 | Rubel, M; Pr | Effect of grape seed extract on remineralization of artificial caries: an in-vitro study.2016 | duplicate | duplicate | 2016 |
| 112 | Giray, FE; D | Resin infiltration technique and fluoride varnish on white spot lesions in children: preliminary findings of a randomized clinical trial.2018 | title/abstract | ineligible intervention | 2018 |
| 113 | ACTRN1260700 | A clinical trial of a tooth cream in the repair of early dental decay.2007 | title/abstract | registry | 2007 |
| 114 | CTRI/2022/06 | Comparing the effect of two remineralizing agents on tooth with white discolouration.2022 | title/abstract | registry | 2022 |
| 115 | Ding, L; He, | In-vitro and in-vivo comparative studies of treatment effects on enamel demineralization during orthodontic therapy: implications for clinical early-intervention strategy.2024 | title/abstract | irrelevant | 2024 |
| 116 | NCT05052775, | EFFICACY OF MORUS ALBA FRUIT EXTRACTS AND CHLORHEXIDINE ON SALIVARY STREPTOCOCCUS MUTANS AND pH LEVELS.2021 | title/abstract | registry | 2021 |
| 117 | Rajendran, R | Remineralization potential of strontium-doped nano-hydroxyapatite dentifrice and casein phosphopeptide-amorphous calcium phosphate cream on white spot lesions in enamel following orthodontic debonding â€“ a randomized controlled trial.2024 | title/abstract | irrelevant | 2024 |
| 118 | CTRI/2023/07 | Effect of two different remineralising agents on white spot lesions.2023 | title/abstract | registry | 2023 |
| 119 | Simon, LS; D | Management of Post Orthodontic White Spot Lesions Using Resin Infiltration and CPP-ACP Materials- A Clinical Study.2022 | title/abstract | irrelevant | 2022 |
| 120 | NCT05771077, | Effect of Poly Amido Amine Dendrimer, Nano-hydroxyapatite.2023 | title/abstract | registry | 2023 |
| 121 | Salah, R; Af | EFFICACY OF NOVEL BIOACTIVE GLASS IN THE TREATMENT OF ENAMEL WHITE SPOT LESIONS: a RANDOMIZED CONTROLLED TRIALâœ°.2022 | title/abstract | irrelevant | 2022 |
| 122 | CiribÃ¨, M; | Efficacy of F-ACP-Containing Dental Mousse in the Remineralization of White Spot Lesions after Fixed Orthodontic Therapy: a Randomized Clinical Trial.2024 | duplicate | duplicate | 2024 |
| 123 | NCT03963791, | Remineralization Effect of Eggshell Powder on Post-orthodontic White Spot Lesions Compared to CPP-ACP.2019 | title/abstract | registry | 2019 |
| 124 | Kleber, CJ; | Effect of remineralizing dentifrice on orthodontic white spots after 3 months.1998 | title/abstract | irrelevant | 1998 |
| 125 | Rajendran, R | Remineralization potential of strontium-doped nano-hydroxyapatite dentifrice and casein phosphopeptide-amorphous calcium phosphate cream on white spot lesions in enamel following orthodontic debonding - a randomized controlled trial.2024 | title/abstract | irrelevant | 2024 |
| 126 | NCT06128434, | Effectiveness of Different Toothpastes in Treating White Spot Lesions.2023 | title/abstract | registry | 2023 |
| 127 | Handa, A; Ch | Effectiveness of Clinpro Tooth CrÃ¨me in comparison with MI Varnish with RECALDENTâ„¢ for treatment of white spot lesions: a randomized controlled trial.2023 | title/abstract | irrelevant | 2023 |
| 128 | NCT04730908, | Dentifrice With Innovative Remineralizing Technology.2021 | title/abstract | registry | 2021 |
| 129 | NCT04357093, | Remineralizing Efficacy of Grape Seed Extract in Management of White Spot Lesion in Post Orthodontic Treated Patients.2020 | title/abstract | registry | 2020 |
| 130 | Heravi, F; A | Effectiveness of MI Paste Plus and Remin Pro on remineralization and color improvement of postorthodontic white spot lesions.2018 | duplicate | duplicate | 2018 |
| 131 | Soltanimehr, | Efficacy of diode and CO2 lasers along with calcium and fluoride-containing compounds for the remineralization of primary teeth.2019 | title/abstract | irrelevant | 2019 |
| 132 | El-Sherif, S | Remineralization Potential Of Pearl Powder Compared To Casein Phosphopeptide Amorphous Calcium Phosphate On Enamel White Spot Lesions (Randomized Clinical Trial).2022 | title/abstract | irrelevant | 2022 |
| 133 | TCTR20200917 | Efficacy of silver diamine fluoride compared to fluoride varnish for arresting non-cavitated lesions on approximal surfaces: a randomized controlled trial.2020 | title/abstract | registry | 2020 |
| 134 | TCTR20210706 | Efficacy of Silver Diamine Fluoride Compared to Fluoride Varnish for Arresting Non-cavitated Lesions on Approximal Surfaces in Permanent Premolars and Molars: a Randomized Controlled Trial.2021 | title/abstract | registry | 2021 |
| 135 | Montenaro, M | Influence of topical fluoride on shear bond strength of orthodontic brackets and enamel white spot lesions formation/ InfluÃªncia do flÃºor tÃ³pico na resistÃªncia ao cisalhamento de braquetes ortodÃ´nticos e na formaÃ§Ã£o de lesÃµes de manchas brancas no esmalte.2021 | title/abstract | irrelevant | 2021 |
| 136 | Shirazi, Mar | The evaluation of shear bond strength of resin-modified glass ionomer cement with the addition of 45S5 bioactive glass using two conventional methods/ La evaluaciÃ³n de la resistencia al cizallamiento del cemento de ionÃ³mero de vidrio modificado con resina con la adiciÃ³n de vidrio bioactivo 45S5 utilizando dos mÃ©todos convencionales.2020 | title/abstract | irrelevant | 2020 |
| 137 | Colodetti, H | AvaliaÃ§Ã£o in vitro de enxaguatÃ³rios experimentais com compostos bioativos na prevenÃ§Ã£o/remineralizaÃ§Ã£o de lesÃµes de mancha branca ativa durante tratamento ortodÃ´ntico/ In vitro evaluation rinses experiments with bioactive compounds in the remineralization of white spot lesions during orthodontic treatment.2016 | title/abstract | irrelevant | 2016 |
| 138 | Cavalli, Van | Influence of fluoride-containing adhesives and bleaching agents on enamel bond strength.2012 | title/abstract | irrelevant | 2012 |
| 139 | Caldeira, Er | Effect of fluoride on tooth erosion around orthodontic brackets.2012 | duplicate | duplicate | 2012 |
| 140 | Hanashiro, F | AvaliaÃ§Ã£o in vitro das superfÃ­cies vestibulares desmineralizadas e restauradas com resina infiltrante/ In vitro evaluation of buccal surfaces demineralized and restored with resin infiltration.2012 | title/abstract | irrelevant | 2012 |
| 141 | Passalini, P | Mechanical properties of one and two-step fluoridated orthodontic resins submitted to different pH cycling regimes.2010 | title/abstract | irrelevant | 2010 |
| 142 | Passalini, P | Preventive effect of fluoridated orthodontic resins subjected to high cariogenic challenges.2010 | title/abstract | irrelevant | 2010 |
| 143 | Oliveira, Mu | AvaliaÃ§Ã£o de nÃ­veis de streptococus mutans na saliva quando usado o tratamento restaurador atraumÃ¡tico em crianÃ§as/ Evaluation of the streptococcus mutans levels in saliva using the atraumatic restorarative treatment at children.2004 | title/abstract | irrelevant | 2004 |
| 144 | Bandeira Lop | Molar-incisor hypomineralization: an umbrella review.2021 | title/abstract | irrelevant | 2021 |
| 145 | Lopes PC, Ca | White spot lesions: diagnosis and treatment - a systematic review.2024 | title/abstract | review | 2024 |
| 146 | Indrapriyadh | Remineralizing potential of CPP-ACP in white spot lesions - A systematic review.2018 | title/abstract | review | 2018 |
| 147 | Xie Z, Yu L, | Comparison of therapies of white spot lesions: a systematic review and network meta-analysis.2023 | title/abstract | review | 2023 |
| 148 | Anil A, Ibra | Nano-Hydroxyapatite (nHAp) in the Remineralization of Early Dental Caries: A Scoping Review.2022 | title/abstract | review | 2022 |
| 149 | Alkilzy M, Q | Biomimetic Enamel Regeneration Using Self-Assembling Peptide P(11)-4.2023 | fulltext | review | 2023 |
| 150 | Peng S, Sang | Bioinspired Anti-demineralization Enamel Coating for Orthodontics.2022 | duplicate | duplicate | 2022 |
| 151 | Linjawi AI. | Sealants and White Spot Lesions in Orthodontics: A Review.2020 | title/abstract | review | 2020 |
| 152 | Dawasaz AA, | Effectiveness of Self-Assembling Peptide (P11-4) in Dental Hard Tissue Conditions: A Comprehensive Review.2022 | fulltext | review | 2022 |
| 153 | Chen H, Liu | Effect of remineralizing agents on white spot lesions after orthodontic treatment: a systematic review.2013 | fulltext | review | 2013 |
| 154 | Liu Y, Ren Z | Therapeutic Strategies Targeting Cariogenic Biofilm Microenvironment.2018 | title/abstract | irrelevant | 2018 |
| 155 | Abuzenada BM | Bionanocomposites in Caries Prevention and Treatment: A Systematic Review.2022 | fulltext | review | 2022 |
| 156 | Fernández-Fe | Enamel remineralization therapies for treating postorthodontic white-spot lesions: A systematic review.2018 | duplicate | duplicate | 2018 |
| 157 | Andrea B, Ca | Biomimetic Action of Zinc Hydroxyapatite on Remineralization of Enamel and Dentin: A Review.2023 | title/abstract | irrelevant | 2023 |
| 158 | Mohamed RN, | Self-assembling peptide P(11)-4 in remineralization of enamel caries - a systematic review of in-vitro studies.2021 | title/abstract | review | 2021 |
| 159 | Lips A, Antu | Salivary protein polymorphisms and risk of dental caries: a systematic review.2017 | title/abstract | irrelevant | 2017 |
| 160 | Sonesson M, | Management of post-orthodontic white spot lesions: an updated systematic review.2017 | title/abstract | irrelevant | 2017 |
| 161 | Pushpalatha | The Anticariogenic Efficacy of Nano Silver Fluoride.2022 | title/abstract | irrelevant | 2022 |
| 162 | Hu H, Feng C | Effectiveness of remineralizing agents in the prevention and reversal of orthodontically induced white spot lesions: a systematic review and network meta-analysis.2020 | duplicate | duplicate | 2020 |
| 163 | Mahmood HT, | Application of New Biomedical Materials in Orthodontic Appliances.2019 | title/abstract | irrelevant | 2019 |
| 164 | Al-Blaihed D | White Spot Lesions in Fixed Orthodontics: A Literature Review on Etiology, Prevention, and Treatment.2024 | title/abstract | irrelevant | 2024 |
| 165 | Li J, Xie X, | Long-term remineralizing effect of casein phosphopeptide-amorphous calcium phosphate (CPP-ACP) on early caries lesions in vivo: a systematic review.2014 | title/abstract | irrelevant | 2014 |
| 166 | Rout T, Pati | Remineralizing potential of Calcium Sucrose Phosphate in white spot lesions: A Systematic Review.2024 | title/abstract | irrelevant | 2024 |
| 167 | Lu X, Qu Y, | Applications of photothermally mediated nanohybrids for white spot lesions in orthodontics.2023 | title/abstract | irrelevant | 2023 |
| 168 | Jardim RN, R | Fabrication and characterization of remineralizing dental composites containing hydroxyapatite nanoparticles.2020 | title/abstract | irrelevant | 2020 |
| 169 | Welk A, Ratz | Effect of self-assembling peptide P(11)-4 on orthodontic treatment-induced carious lesions.2020 | duplicate | duplicate | 2020 |
| 170 | Wahba N, Sch | Preventing and Arresting Primary Tooth Enamel Lesions Using Self- Assembling Peptide P(11)-4 In Vitro.2022 | fulltext | ineligible population | 2022 |
| 171 | Ferreira CJ, | Antibacterial and Remineralizing Fillers in Experimental Orthodontic Adhesives.2019 | fulltext | ineligible intervention | 2019 |
| 172 | Bian C, Guo | New generation of orthodontic devices and materials with bioactive capacities to improve enamel demineralization.2024 | title/abstract | irrelevant | 2024 |
| 173 | Rm VR, Singh | Salivary physicochemical characteristics and antimicrobial human peptide among Indian children with dental caries.2023 | title/abstract | irrelevant | 2023 |
| 174 | da Silva Mei | Fabrication and characterization of remineralizing dental composites containing calcium type pre-reacted glass-ionomer (PRG-Ca) fillers.2021 | title/abstract | irrelevant | 2021 |
| 175 | Ludovichetti | Prevention of White Spot Lesions Induced by Fixed Orthodontic Therapy: A Literature Review.2025 | title/abstract | review | 2025 |
| 176 | Ma Y, Zhang | Novel multifunctional dental cement to prevent enamel demineralization near orthodontic brackets.2017 | duplicate | duplicate | 2017 |
| 177 | Kucuk EB, Ma | Microcomputed tomography evaluation of white spot lesion remineralization with various procedures.2016 | title/abstract | irrelevant | 2016 |
| 178 | Shah SA, Sha | Evaluation of Remineralizing Capacity of Tricalcium Phosphate, Nano-Hydroxyapatite and Ozone Remineralizing Agents on the Artificial Carious Lesion.2024 | title/abstract | irrelevant | 2024 |
| 179 | Simon LS, Da | Management of Post Orthodontic White Spot Lesions Using Resin Infiltration and CPP-ACP Materials- A Clinical Study.2022 | duplicate | duplicate | 2022 |
| 180 | Fernandes GL | Antibacterial, cytotoxic and mechanical properties of a orthodontic cement with phosphate nano-sized and phosphorylated chitosan: An in vitro study.2024 | title/abstract | irrelevant | 2024 |
| 181 | Ahmed T, Far | Effect of re-mineralizing surface treatment on the shear bond strength of orthodontic bracket: A systematic review and meta-analysis of in-vitro studies.2023 | title/abstract | review | 2023 |
| 182 | Knaup T, Kor | Effect of the caries-protective self-assembling peptide P11-4 on shear bond strength of metal brackets.2021 | fulltext | ineligible outcome | 2021 |
| 183 | Bakhsh TA, B | Novel evaluation and treatment techniques for white spot lesions. An in vitro study.2017 | duplicate | duplicate | 2017 |
| 184 | Hamdi K, Els | Remineralization and anti-demineralization effect of orthodontic adhesives on enamel surrounding orthodontic brackets: a systematic review of in vitro studies.2024 | title/abstract | review | 2024 |
| 185 | Taha AA, Pat | The effect of bioactive glasses on enamel remineralization: A systematic review.2017 | title/abstract | review | 2017 |
| 186 | Aref NS, Als | Surface topography and spectrophotometric assessment of white spot lesions restored with nano-hydroxyapatite-containing universal adhesive resin: an in-vitro study.2023 | title/abstract | irrelevant | 2023 |
| 187 | Hoxie AM, Ne | Microcomputed Tomography Mineral Density Profile as Reference Standard for Early Carious Lesion Activity Assessment.2023 | title/abstract | irrelevant | 2023 |
| 188 | Liu Y, Zhang | Antibacterial and remineralizing orthodontic adhesive containing quaternary ammonium resin monomer and amorphous calcium phosphate nanoparticles.2018 | title/abstract | irrelevant | 2018 |
| 189 | Lopatiene K, | Prevention and Treatment of White Spot Lesions During and After Treatment with Fixed Orthodontic Appliances: a Systematic Literature Review.2016 | title/abstract | review | 2016 |
| 190 | Pliska BT, W | Treatment of white spot lesions with ACP paste and microabrasion.2012 | title/abstract | irrelevant | 2012 |
| 191 | Güven E, Ede | Remineralization of post-orthodontic white spot lesions with a fluoride varnish and a self-assembling P 11 - 4 peptides: a prospective in-vivo-study.2024 | fulltext | included | 2024 |
| 192 | Butera A, Pa | Biomimetic hydroxyapatite paste for molar-incisor hypomineralization: A randomized clinical trial.2023 | title/abstract | irrelevant | 2023 |
| 193 | Wierichs RJ, | Short-term efficacy of caries resin infiltration during treatment with orthodontic fixed appliances. A randomized controlled trial.2023 | duplicate | duplicate | 2023 |
| 194 | Pires PM, Ro | Bioactive Restorative Materials Applied over Coronal Dentine-A Bibliometric and Critical Review.2023 | title/abstract | review | 2023 |
| 195 | Garcia R, Bo | Progress in Early Childhood Caries and Opportunities in Research, Policy, and Clinical Management.2015 | title/abstract | review | 2015 |
| 196 | Kohda N, Iij | Inhibition of enamel demineralization and bond-strength properties of bioactive glass containing 4-META/MMA-TBB-based resin adhesive.2015 | duplicate | duplicate | 2015 |
| 197 | Kawamura N, | Wear characteristics and inhibition of enamel demineralization by resin-based coating materials.2017 | duplicate | duplicate | 2017 |
| 198 | Tahmasbi S, | Prevention of white spot lesions using three remineralizing agents: An in vitro comparative study.2019 | title/abstract | irrelevant | 2019 |
| 199 | Chen Y, Chen | Bio-inspired nanocomposite coatings on orthodontic archwires with corrosion resistant and antibacterial properties.2023 | duplicate | duplicate | 2023 |
| 200 | Abbassy MA, | Characterization of a novel enamel sealer for bioactive remineralization of white spot lesions.2021 | title/abstract | irrelevant | 2021 |
| 201 | Martins C, B | Salivary proteins as a biomarker for dental caries--a systematic review.2013 | title/abstract | review | 2013 |
| 202 | Zhang W, Vaz | Decellularized Tooth Bud Scaffolds for Tooth Regeneration.2017 | title/abstract | irrelevant | 2017 |
| 203 | Alsubhi H, G | A Comparison between Two Different Remineralizing Agents against White Spot Lesions: An In Vitro Study.2021 | duplicate | duplicate | 2021 |
| 204 | Ghaly YS, El | Effect of self-assembling peptide and other remineralizing agents on preventing initial enamel lesions around orthodontic brackets: An in vitro comparative study.2023 | fulltext | included | 2023 |
| 205 | Butera A, Ma | Evaluation of Children Caries Risk Factors: A Narrative Review of Nutritional Aspects, Oral Hygiene Habits, and Bacterial Alterations.2022 | title/abstract | irrelevant | 2022 |
| 206 | Chatzidimitr | The role of hydroxyapatite-based, fluoride-free toothpastes on the prevention and the remineralization of initial caries lesions: A systematic review and meta-analysis.2025 | title/abstract | irrelevant | 2025 |
| 207 | Halusic AM, | Calcium and magnesium levels in primary tooth enamel and genetic variation in enamel formation genes.2014 | title/abstract | irrelevant | 2014 |
| 208 | Ibrahim AI, | Enamel conservation orthodontic paradigm via treatment with newly developed remineralizing calcium-phosphate etchant pastes.2025 | duplicate | duplicate | 2025 |
| 209 | Nalawade VA, | Effectiveness of Various Remineralizing Agents on White Spot Lesions after Orthodontic Treatment: A Comparative Study.2021 | title/abstract | irrelevant | 2021 |
| 210 | Rahmanpanah | Evaluation of shear bond strength and enamel remineralizing effect of experimental orthodontic composite containing nano-hydroxyapatite: An in vitro study.2023 | title/abstract | irrelevant | 2023 |
| 211 | Manfred L, C | A novel biomimetic orthodontic bonding agent helps prevent white spot lesions adjacent to brackets.2013 | title/abstract | irrelevant | 2013 |
| 212 | Ogaard B, Rø | Orthodontic appliances and enamel demineralization. Part 2. Prevention and treatment of lesions.1988 | duplicate | duplicate | 1988 |
| 213 | Shetty SS, N | Remineralization Potential of a Novel Biomimetic Material (Self-assembling Peptide P(11-4)) on Early Enamel Caries: An In Vitro Study.2023 | title/abstract | irrelevant | 2023 |
| 214 | Rangappa KG, | Remineralization Potential of a Combination of Chitosan with Nanohydroxyapatite and a Self-assembling Peptide with Nanohydroxyapatite: An In Vitro Study.2022 | title/abstract | irrelevant | 2022 |
| 215 | Hua F, Yan J | In vitro remineralization of enamel white spot lesions with a carrier-based amorphous calcium phosphate delivery system.2020 | title/abstract | irrelevant | 2020 |
| 216 | Zhou L, Li Q | A Novel Strategy for Caries Management: Constructing an Antibiofouling and Mineralizing Dual-Bioactive Tooth Surface.2021 | title/abstract | irrelevant | 2021 |
| 217 | Balakrishnan | Enamel Remineralization Efficacy of Coconut Milk and Lyophilized Coconut Extract in Different Concentrations on Demineralized Enamel Surfaces: An In-Vitro Study.2023 | title/abstract | irrelevant | 2023 |
| 218 | Gohar RAAEG, | Evaluation of the remineralizing effect of biomimetic self-assembling peptides in post-orthodontic white spot lesions compared to fluoride-based delivery systems: randomized controlled trial.2023 | fulltext | included | 2023 |
| 219 | Uy E, Ekamba | Remineralization Potential of Calcium and Phosphate-based Agents and Their Effects on Bonding of Orthodontic Brackets.2019 | title/abstract | irrelevant | 2019 |
| 220 | Guo M, Yang | Inhibitory effects of Stevioside on Streptococcus mutans and Candida albicans dual-species biofilm.2023 | title/abstract | irrelevant | 2023 |
| 221 | Altmann AS, | In vitro antibacterial and remineralizing effect of adhesive containing triazine and niobium pentoxide phosphate inverted glass.2017 | title/abstract | irrelevant | 2017 |
| 222 | Jablonski-Mo | Impact of self-assembling peptides in remineralisation of artificial early enamel lesions adjacent to orthodontic brackets.2020 | duplicate | duplicate | 2020 |
| 223 | Basiri T, Jo | Duplicated or Hybridized Peptide Functional Domains Promote Oral Homeostasis.2017 | title/abstract | irrelevant | 2017 |
| 224 | Mollabashi V | Evaluation of Nano TiO(2) Modified Orthodontic Composite Effects on S. mutans Population and Enamel Demineralization in Fixed Orthodontic Patients; a Split Mouth Randomized Controlled Clinical Trial.2023 | title/abstract | irrelevant | 2023 |
| 225 | Cagetti MG, | Efficacy of HAF toothpastes in primary and permanent dentitions. A 2-years triple-blind RCT.2022 | title/abstract | irrelevant | 2022 |
| 226 | Splieth CH, | Caries-preventive and remineralizing effect of fluoride gel in orthodontic patients after 2 years.2012 | title/abstract | irrelevant | 2012 |
| 227 | Bakry AS, Ab | Increasing the efficiency of CPP-ACP to remineralize enamel white spot lesions.2018 | title/abstract | irrelevant | 2018 |
| 228 | Aggarwal P, | Comparison of micromorphological changes in enamel using SEM analysis after conventional and erbium, chromium:yttrium, scandium, gallium, and garnet hard-tissue laser fissurotomy: An in vitro study.2023 | title/abstract | irrelevant | 2023 |
| 229 | Butera A, Ga | Home Oral Care with Biomimetic Hydroxyapatite vs. Conventional Fluoridated Toothpaste for the Remineralization and Desensitizing of White Spot Lesions: Randomized Clinical Trial.2022 | title/abstract | irrelevant | 2022 |
| 230 | Aref NS, Alr | Casein phosphopeptide amorphous calcium phosphate and universal adhesive resin as a complementary approach for management of white spot lesions: an in-vitro study.2022 | title/abstract | irrelevant | 2022 |
| 231 | Bhat DV, Awc | Evaluation of Remineralizing Potential of CPP-ACP, CPP-ACP + F and β TCP + F and Their Effect on Microhardness of Enamel Using Vickers Microhardness Test: An In Vitro Study.2022 | duplicate | duplicate | 2022 |
| 232 | Chin MY, San | Fluoride release and cariostatic potential of orthodontic adhesives with and without daily fluoride rinsing.2009 | duplicate | duplicate | 2009 |
| 233 | Peng S, Guan | Efficacy of peptide-based enamel coatings in the prevention of demineralization using fixed orthodontic brackets in a rat model.2024 | title/abstract | irrelevant | 2024 |
| 234 | Fathy Abo-El | Evaluation of the remineralizing effect of the chicken eggshell paste after removal of the fixed orthodontic appliance: An in vitro study.2024 | title/abstract | irrelevant | 2024 |
| 235 | Ben Mohimd H | Enamel protection after stripping procedures: An in vivo study.2019 | title/abstract | irrelevant | 2019 |
| 236 | Hamed S, Abd | Fluoride-amorphous calcium phosphate and biomimetic nano-hydroxyapatite for enamel remineralization: An in-vitro study of surface microhardness and composition.2024 | title/abstract | irrelevant | 2024 |
| 237 | Jablonski-Mo | Randomised in situ clinical trial investigating self-assembling peptide matrix P11-4 in the prevention of artificial caries lesions.2019 | title/abstract | irrelevant | 2019 |
| 238 | Moraes SM, K | Effectiveness of Fluoride Varnishes for White Spot Lesion Prevention and Remineralization during Orthodontic Treatment: A Randomized Controlled Trial.2024 | duplicate | duplicate | 2024 |
| 239 | Rana N, Sing | A comparative evaluation of penetration depth and surface microhardness of Resin Infiltrant, CPP-ACPF and Novamin on enamel demineralization after banding: an in vitro study.2021 | duplicate | duplicate | 2021 |
| 240 | Lena Sezici | Comparative evaluation of fluoride varnishes, self-assembling peptide-based remineralization agent, and enamel matrix protein derivative on artificial enamel remineralization in vitro.2021 | duplicate | duplicate | 2021 |
| 241 | Dai Z, Liu M | Effects of Fluoride and Calcium Phosphate Materials on Remineralization of Mild and Severe White Spot Lesions.2019 | title/abstract | irrelevant | 2019 |
| 242 | Yu J, Qin D, | THE EFFECTS OF PREVENTIVE INTERVENTIONS FOR ORTHODONTICALLY INDUCED WHITE SPOT LESIONS: AN UMBRELLA REVIEW AND EVIDENCE MAPPING.2025 | title/abstract | irrelevant | 2025 |
| 243 | Seifi M, Esk | Investigation of mechanical properties, remineralization, antibacterial effect, and cellular toxicity of composite orthodontic adhesive combined with silver-containing nanostructured bioactive glass.2024 | title/abstract | irrelevant | 2024 |
| 244 | Songsiriprad | Sodium fluoride mouthrinse used twice daily increased incipient caries lesion remineralization in an in situ model.2014 | duplicate | duplicate | 2014 |
| 245 | Singh S, Sin | Effects of various remineralizing agents on the outcome of post-orthodontic white spot lesions (WSLs): a clinical trial.2016 | duplicate | duplicate | 2016 |
| 246 | Jablonski-Mo | Efficacy of the self-assembling peptide P11-4 in constructing a remineralization scaffold on artificially-induced enamel lesions on smooth surfaces.2014 | duplicate | duplicate | 2014 |
| 247 | Thierens LAM | The in vitro remineralizing effect of CPP-ACP and CPP-ACPF after 6 and 12 weeks on initial caries lesion.2019 | title/abstract | ineligible intervention | 2019 |
| 248 | Qin Q, Yuan | A pH-sensitive, renewable invisible orthodontic aligners coating manipulates antibacterial and in situ remineralization functions to combat enamel demineralization.2024 | title/abstract | irrelevant | 2024 |
| 249 | Pavethynath | Evaluation of Prevention of Initial Enamel Lesions around Orthodontic Brackets by Using Different Remineralizing Agents: An Original Research.2024 | duplicate | duplicate | 2024 |
| 250 | Samuel SR, D | Effect of ozone to remineralize initial enamel caries: in situ study.2016 | title/abstract | irrelevant | 2016 |
| 251 | Loewe MF, Do | Biofilm volume and acidification within initial biofilms formed in situ on buccally and palatally exposed bracket material.2024 | duplicate | duplicate | 2024 |
| 252 | Valente MT, | Acquired Enamel Pellicle Engineered Peptides: Effects on Hydroxyapatite Crystal Growth.2018 | title/abstract | irrelevant | 2018 |
| 253 | Zhang N, Zha | Novel protein-repellent and biofilm-repellent orthodontic cement containing 2-methacryloyloxyethyl phosphorylcholine.2016 | title/abstract | irrelevant | 2016 |
| 254 | Heravi F, Ah | Effectiveness of MI Paste Plus and Remin Pro on remineralization and color improvement of postorthodontic white spot lesions.2018 | duplicate | duplicate | 2018 |
| 255 | Sain S, Hegd | Remineralization of Enamel Using Topical Agents among Patients with Orthodontic Brackets: In Vivo and In Vitro Randomized Control Trial.2024 | title/abstract | irrelevant | 2024 |
| 256 | Giannetti L, | "Superficial infiltration to treat white hypomineralized defects of enamel: clinical trial with 12-month follow-up.2018 | title/abstract | irrelevant | 2018 |
| 257 | Gao SS, Amar | Global Oral Health Policies and Guidelines: Using Silver Diamine Fluoride for Caries Control.2021 | title/abstract | irrelevant | 2021 |
| 258 | Eldeeb AI, T | Effect of Biomin F toothpaste and Diode laser on remineralization of white spot lesions (in vitro study).2024 | title/abstract | irrelevant | 2024 |
| 259 | Scribante A, | Biomimetic Effect of Nano-Hydroxyapatite in Demineralized Enamel before Orthodontic Bonding of Brackets and Attachments: Visual, Adhesion Strength, and Hardness in In Vitro Tests.2020 | title/abstract | irrelevant | 2020 |
| 260 | Balakrishnan | Three-dimensional analysis of the potency of Cocos nucifera in demineralizing the artificial enamel carious lesion: a micro-CT study.2024 | title/abstract | irrelevant | 2024 |
| 261 | Yadav P, Des | A comparative quantitative & qualitative assessment in orthodontic treatment of white spot lesion treated with 3 different commercially available materials - In vitro study.2019 | duplicate | irrelevant | 2019 |
| 262 | Munjal D, Ga | Assessment of White Spot Lesions and In-Vivo Evaluation of the Effect of CPP-ACP on White Spot Lesions in Permanent Molars of Children.2016 | duplicate | ineligible intervention | 2016 |
| 263 | Ogihara S, T | Efficacy of forced eruption/enamel matrix derivative with freeze-dried bone allograft or with demineralized freeze-dried bone allograft in infrabony defects: A randomized trial.2015 | duplicate | duplicate | 2015 |
| 264 | Bakry AS, Al | In-vitro comparative study for three different strategies to treat enamel demineralized white spot lesion.2025 | duplicate | irrelevant | 2025 |
| 265 | Senthilkumar | Assessment of the Additive Effect of Remineralizing Agents in Combination With Fluoride Releasing Adhesives in the Prevention of Enamel Decalcification in Orthodontic Patients: An In Vitro Study.2024 | title/abstract | irrelevant | 2024 |
| 266 | Ebrahimi M, | The effects of three remineralizing agents on regression of white spot lesions in children: A two-week, single-blind, randomized clinical trial.2017 | title/abstract | irrelevant | 2017 |
| 267 | Shirazi M, T | Effect of addition of bioactive glass to resin modified glass ionomer cement on enamel demineralization under orthodontic brackets.2019 | title/abstract | irrelevant | 2019 |
| 268 | Ciribè M, Ci | Efficacy of F-ACP-Containing Dental Mousse in the Remineralization of White Spot Lesions after Fixed Orthodontic Therapy: A Randomized Clinical Trial.2024 | duplicate | duplicate | 2024 |
| 269 | Thorat SU, B | Evaluating the Remineralizing Effects of Calcium Sucrose Phosphate and Casein Phosphopeptide-Amorphous Calcium Phosphate Toothpastes on Artificial Carious Lesions Using Micro-computed Tomography: An In Vitro Investigation.2024 | title/abstract | irrelevant | 2024 |
| 270 | Ghadirian H, | The Effect of Remineralizing Agents With/Without CO(2) Laser Irradiation on Structural and Mechanical Properties of Enamel and its Shear Bond Strength to Orthodontic Brackets.2020 | title/abstract | irrelevant | 2020 |
| 271 | Majithia U, | Comparative evaluation of application of different fluoride varnishes on artificial early enamel lesion: An in vitro study.2016 | title/abstract | irrelevant | 2016 |
| 272 | Bakr NM, Moh | Comparative evaluation of the remineralizing potential of Salvadora persica and probiotic yogurt on incipient enamel lesions: An ex-vivo study.2024 | title/abstract | irrelevant | 2024 |
| 273 | Yadahalli RP | Comparative Evaluation of Efficacy of Self-Assembling Peptide (P11-4), Bioactive Glass, and Arginine Bicarbonate Remineralizing Agents on Simulated Carious Lesion.2024 | fulltext | ineligible population | 2024 |
| 274 | Seminario AL | Salivary Cathelicidin (LL-37) in Children and Adolescents Living with HIV.2024 | title/abstract | irrelevant | 2024 |
| 275 | Bansal K, Ba | Remineralizing efficacy of Calcarea Fluorica tablets on the artificial carious enamel lesions using scanning electron microscope and surface microhardness testing: in vivo study.2014 | title/abstract | irrelevant | 2014 |
| 276 | Ahrari F, Mo | The effect of diode laser irradiation associated with photoabsorbing agents containing remineralizing materials on microhardness, morphology and chemical structure of early enamel caries.2018 | title/abstract | irrelevant | 2018 |
| 277 | Nandhini G, | Comparative analysis of remineralizing efficacy of strontium-doped bioactive glass, BioMin, and NovaMin containing dentifrice on artificial white spot lesions after chlorhexidine pretreatment - An in vitro study.2024 | title/abstract | irrelevant | 2024 |
| 278 | Reis DP, Fil | Remineralizing potential of dental composites containing silanized silica-hydroxyapatite (Si-HAp) nanoporous particles charged with sodium fluoride (NaF).2019 | title/abstract | irrelevant | 2019 |
| 279 | Yassaei S, A | Efficacy of erbium-doped yttrium aluminum garnet laser with casein phosphopeptide amorphous calcium phosphate with and without fluoride for remineralization of white spot lesions around orthodontic brackets.2018 | duplicate | duplicate | 2018 |
| 280 | Umer D, Yiu | Effect of a novel quaternary ammonium silane on dentin protease activities.2017 | title/abstract | irrelevant | 2017 |
| 281 | Bhongsatiern | Adjunctive use of fluoride rinsing and brush-on gel increased incipient caries-like lesion remineralization compared with fluoride toothpaste alone in situ.2019 | duplicate | duplicate | 2019 |
| 282 | Beerens MW, | Long-term remineralizing effect of MI Paste Plus on regression of early caries after orthodontic fixed appliance treatment: a 12-month follow-up randomized controlled trial.2018 | duplicate | duplicate | 2018 |
| 283 | Najjaran H, | Effect of Diode Laser and Fluoride Varnish on Microhardness of enamel: An In Vitro Study.2024 | title/abstract | irrelevant | 2024 |
| 284 | Danisman H, | Evaluation of the efficacy of P11-4 and CCP-ACPF in the prevention and treatment of white spot lesions: a multi-technique approach.2025 | fulltext | ineligible population | 2025 |
| 285 | Alam MK, Zhe | Synthetic antigen-binding fragments (Fabs) against S. mutans and S. sobrinus inhibit caries formation.2018 | title/abstract | irrelevant | 2018 |
| 286 | Bailey DL, A | Regression of post-orthodontic lesions by a remineralizing cream.2009 | duplicate | duplicate | 2009 |
| 287 | Naha PC, Liu | Dextran-Coated Iron Oxide Nanoparticles as Biomimetic Catalysts for Localized and pH-Activated Biofilm Disruption.2019 | title/abstract | irrelevant | 2019 |
| 288 | Damle SG, Be | Effect of dentifrices on their remineralizing potential in artificial carious lesions: An in situ study.2016 | title/abstract | irrelevant | 2016 |
| 289 | Wang JX, Yan | Clinical evaluation of remineralization potential of casein phosphopeptide amorphous calcium phosphate nanocomplexes for enamel decalcification in orthodontics.2012 | title/abstract | irrelevant | 2012 |
| 290 | Rajendran R, | Evaluation of remineralization potential and cytotoxicity of a novel strontium-doped nanohydroxyapatite paste: An in vitro study.2020 | title/abstract | irrelevant | 2020 |
| 291 | Wattanarat O | Enhancement of salivary human neutrophil peptide 1-3 levels by probiotic supplementation.2015 | title/abstract | irrelevant | 2015 |
| 292 | Bergstrand F | A review on prevention and treatment of post-orthodontic white spot lesions - evidence-based methods and emerging technologies.2011 | title/abstract | irrelevant | 2011 |
| 293 | Caldeira EM, | Effect of fluoride on tooth erosion around orthodontic brackets.2012 | title/abstract | irrelevant | 2012 |
| 294 | Burbank BD, | Ion release, fluoride charge of and adhesion of an orthodontic cement paste containing microcapsules.2016 | duplicate | duplicate | 2016 |
| 295 | Brown ML, Da | Ion release from a novel orthodontic resin bonding agent for the reduction and/or prevention of white spot lesions. An in vitro study.2011 | title/abstract | irrelevant | 2011 |
| 296 | Agha N, Qasi | EFFECTS OF SESAMUM INDICUM (SESAME) OIL IN REMINERALIZING OF WHITE SPOT LESIONS INDUCED AFTER BRACKET DEBONDING: AN IN VITRO STUDY.2024 | duplicate | duplicate | 2024 |
| 297 | Bansal K, Ga | In vivo remineralization of artificial enamel carious lesions using a mineral-enriched mouthrinse and a fluoride dentifrice: a polarized light microscopic comparative evaluation.2010 | duplicate | duplicate | 2010 |
| 298 | Bakry AS, Ab | A Novel Fluoride Containing Bioactive Glass Paste is Capable of Re-Mineralizing Early Caries Lesions.2018 | duplicate | duplicate | 2018 |
| 299 | Jagga U, Pau | Comparative Evaluation of Remineralizing Effect of Novamin and Tricalcium Phosphate on Artificial Caries: An in vitro Study.2018 | title/abstract | irrelevant | 2018 |
| 300 | Obeid AT, Ga | Effects of hybrid inorganic-organic nanofibers on the properties of enamel resin infiltrants - An in vitro study.2022 | title/abstract | irrelevant | 2022 |
| 301 | Linton JL. | Quantitative measurements of remineralization of incipient caries.1996 | title/abstract | irrelevant | 1996 |
| 302 | Iijima M, It | Effects of the addition of fluoride to a 4-META/MMA-TBB-based resin adhesive on fluoride release, acid resistance of enamel and shear bond strength in vitro.2013 | duplicate | duplicate | 2013 |
| 303 | Pourhajibagh | Quorum quenching of Streptococcus mutans via the nano-quercetin-based antimicrobial photodynamic therapy as a potential target for cariogenic biofilm.2022 | title/abstract | irrelevant | 2022 |
| 304 | Jayarajan J, | Efficacy of CPP-ACP and CPP-ACPF on enamel remineralization - an in vitro study using scanning electron microscope and DIAGNOdent.2011 | title/abstract | irrelevant | 2011 |
| 305 | Verma P, Mut | Bionic effects of nano hydroxyapatite dentifrice on demineralised surface of enamel post orthodontic debonding: in-vivo split mouth study.2021 | duplicate | duplicate | 2021 |
| 306 | Haerian A, Y | In Vitro Efficacy of Tricalcium Phosphate and Casein Phosphopeptide Amorphous Calcium Phosphate Fluoride for Remineralization of Enamel White Spot Lesions.2024 | title/abstract | ineligible intervention | 2024 |
| 307 | Kleber CJ, M | Treatment of orthodontic white spot lesions with a remineralizing dentifrice applied by toothbrushing or mouth trays.1999 | title/abstract | ineligible intervention | 1999 |
| 308 | Singer L, Ka | A novel stable biomimetic adhesive coating for functionalization of orthodontic brackets against bacterial colonization and white spot lesions.2025 | title/abstract | ineligible intervention | 2025 |
| 309 | Klimaitė G, | The Efficacy of Remineralizing Materials on Artificial Enamel Lesions: An In Vitro Study.2025 | title/abstract | irrelevant | 2025 |
| 310 | Fırıncıoğull | Effects of Fluoride-containing Biomimetic Agents on Remineralization and Color Change of Enamel White Spot Lesions: An In Vitro Study.2025 | title/abstract | ineligible population | 2025 |
| 311 | Dixit A, Mam | Analysis of Remineralization Potential of Three Different Remineralizing Pastes on Demineralized Enamel: A Comparative Study.2021 | title/abstract | irrelevant | 2021 |
| 312 | Baeshen HA, | Effect of fluoridated chewing sticks (Miswaks) on white spot lesions in postorthodontic patients.2011 | duplicate | duplicate | 2011 |
| 313 | Dewani N, Ka | Effect of casein phosphopeptide-amorphous calcium phosphate as a remineralizing agent - An In Vivo study.2019 | title/abstract | ineligible intervention | 2019 |
| 314 | Aboulnaga MA | Evaluation of Remineralization Potential of Remin Pro Forte vs Remin Pro on White Spot Lesions: A Randomized Clinical Trial.2022 | duplicate | duplicate | 2022 |
| 315 | Wattanarat O | Significant elevation of salivary human neutrophil peptides 1-3 levels by probiotic milk in preschool children with severe early childhood caries: a randomized controlled trial.2021 | title/abstract | irrelevant | 2021 |
| 316 | Iijima M, Is | Effects of pastes containing ion-releasing particles on dentin remineralization.2019 | title/abstract | irrelevant | 2019 |
| 317 | Agarwal A, P | Effect of fluoridated toothpaste on white spot lesions in postorthodontic patients.2013 | title/abstract | irrelevant | 2013 |
| 318 | Reddy VS, Su | A comparative evaluation of human enamel remineralization ability of biomimetic nacre against casein phosphopeptide-amorphous calcium phosphate: An in vitro study.2024 | title/abstract | irrelevant | 2024 |
| 319 | Butera A, Ga | Home Oral Care Domiciliary Protocol for the Management of Dental Erosion in Rugby Players: A Randomized Clinical Trial.2022 | title/abstract | irrelevant | 2022 |
| 320 | Kemény A, Fa | The effect of various bulk filling techniques on the mechanical and structural characteristics of class I biomimetic composite dental fillings.2025 | title/abstract | irrelevant | 2025 |
| 321 | Helal MB, Sh | Comparing the remineralization potential of undemineralized dentin powder versus chicken eggshell powder on artificially induced initial enamel carious lesions: an in-vitro investigation.2024 | title/abstract | irrelevant | 2024 |
| 322 | Pretty IA, P | The in vitro detection of early enamel de- and re-mineralization adjacent to bonded orthodontic cleats using quantitative light-induced fluorescence.2003 | title/abstract | irrelevant | 2003 |
| 323 | Somasundaram | Protective potential of casein phosphopeptide amorphous calcium phosphate containing paste on enamel surfaces.2013 | title/abstract | irrelevant | 2013 |
| 324 | Joshi S, Vai | A Comparative Evaluation of Arginine Complex Combined With Flouride and Two Standard Non-Fluoridated Remineralizing Agents: An In Vitro Study.2024 | duplicate | duplicate | 2024 |
| 325 | Niazi FH, Al | The Adhesive Strength and Degree of Conversion of Remineralizing Hydroxyapatite, Amorphous Calcium Phosphate, and Cerium Oxide Fillers in Experimental Adhesive: A SEM and EDX Analysis.2025 | title/abstract | irrelevant | 2025 |
| 326 | Bollineni S, | Role of fluoridated carbamide peroxide whitening gel in the remineralization of demineralized enamel: An in vitro study.2014 | title/abstract | irrelevant | 2014 |
| 327 | Hong SC, Lee | Micro-computed tomographic evaluation of the effect of fluoride agents on white spot lesions: An in vitro study.2022 | title/abstract | irrelevant | 2022 |
| 328 | Heshmat H, B | The effect of recommending a CPP-ACPF product on salivary and plaque pH levels in orthodontic patients: a randomized cross-over clinical trial.2014 | title/abstract | irrelevant | 2014 |
| 329 | Langhorst SE | In vitro remineralization of enamel by polymeric amorphous calcium phosphate composite: quantitative microradiographic study.2009 | title/abstract | irrelevant | 2009 |
| 330 | Nozari A, Aj | Impact of Nano Hydroxyapatite, Nano Silver Fluoride and Sodium Fluoride Varnish on Primary Teeth Enamel Remineralization: An In Vitro Study.2017 | title/abstract | irrelevant | 2017 |
| 331 | Passalini P, | Mechanical properties of one and two-step fluoridated orthodontic resins submitted to different pH cycling regimes.2010 | duplicate | duplicate | 2010 |
| 332 | Scribante A, | Clinical and Technological Evaluation of the Remineralising Effect of Biomimetic Hydroxyapatite in a Population Aged 6 to 18 Years: A Randomized Clinical Trial.2025 | title/abstract | irrelevant | 2025 |
| 333 | Liu Y, Kames | Topical delivery of low-cost protein drug candidates made in chloroplasts for biofilm disruption and uptake by oral epithelial cells.2016 | title/abstract | irrelevant | 2016 |
| 334 | Ajaj MT, Al- | Effect of different acid etchants on the remineralization process of white-spot lesions: An in vitro study.2020 | title/abstract | irrelevant | 2020 |
| 335 | Mayne RJ, Co | In-vitro study of the effect of casein phosphopeptide amorphous calcium fluoride phosphate on iatrogenic damage to enamel during orthodontic adhesive removal.2011 | duplicate | duplicate | 2011 |
| 336 | Wan Hassan W | A 12-months randomized clinical trial comparing fluoride-based remineralising protocols on post-orthodontic initial caries lesions.2025 | duplicate | duplicate | 2025 |
| 337 | Patil N, Jaw | In vitro caries-preventive effect of fluoridated orthodontic resins against cariogenic challenge stimulation.2012 | title/abstract | irrelevant | 2012 |
| 338 | Catros S, Po | Collagen fibrils of human acellular extrinsic fiber cementum.2008 | title/abstract | irrelevant | 2008 |
| 339 | Bandekar S, | The Remineralization Potential of Fluoride, Casein Phosphopeptide-Amorphous Calcium Phosphate, and Chicken Eggshell on Enamel Lesions: An In Vitro Study.2025 | title/abstract | irrelevant | 2025 |
| 340 | Mohanty P, P | An in Vitro Evaluation of Remineralization Potential of Novamin(®) on Artificial Enamel Sub-Surface Lesions Around Orthodontic Brackets Using Energy Dispersive X-Ray Analysis (EDX).2014 | title/abstract | irrelevant | 2014 |
| 341 | Velo MMAC, M | Profile of high-fluoride toothpastes combined or not with functionalized tri-calcium phosphate on root dentin caries control: An in vitro study.2018 | title/abstract | irrelevant | 2018 |
| 342 | Passalini P, | Preventive effect of fluoridated orthodontic resins subjected to high cariogenic challenges.2010 | duplicate | duplicate | 2010 |
| 343 | Malterud MI. | Minimally invasive restorative dentistry: a biomimetic approach.2006 | title/abstract | irrelevant | 2006 |
| 344 | Brignardello | There are probably no differences in arrest of white-spot lesions and plaque composition between remineralizing and nonfluoridated toothpastes.2018 | title/abstract | irrelevant | 2018 |
| 345 | Marquezan M, | Resistance to degradation of bonded restorations to simulated caries-affected primary dentin.2010 | title/abstract | irrelevant | 2010 |
| 346 | Casals E, Bo | Anticaries potential of commercial dentifrices as determined by fluoridation and remineralization efficiency.2007 | duplicate | duplicate | 2007 |
| 347 | Singer L.; K | A novel stable biomimetic adhesive coating for functionalization of orthodontic brackets against bacterial colonization and white spot lesions.2025 | duplicate | duplicate | 2025 |
| 348 | Nandhini G.; | Comparative analysis of remineralizing efficacy of strontium‑doped bioactive glass, BioMin, and NovaMin containing dentifrice on artificial white spot lesions after chlorhexidine pretreatment ‑ An in vitro study.2024 | fulltext | ineligible population | 2024 |
| 349 | Rawal K.; Ma | Evaluation of efficacy of various remineralizing agents on artificially demineralized human enamel – An in-vitro study.2023 | title/abstract | irrelevant | 2023 |
| 350 | Aref N.S.; A | Surface topography and spectrophotometric assessment of white spot lesions restored with nano-hydroxyapatite-containing universal adhesive resin: an in-vitro study.2023 | duplicate | duplicate | 2023 |
| 351 | Lu X.; Qu Y. | Applications of photothermally mediated nanohybrids for white spot lesions in orthodontics.2023 | duplicate | duplicate | 2023 |
| 352 | Seifi M.; Es | Investigation of mechanical properties, remineralization, antibacterial effect, and cellular toxicity of composite orthodontic adhesive combined with silver-containing nanostructured bioactive glass.2024 | duplicate | duplicate | 2024 |
| 353 | Jablonski-Mo | Randomised in situ clinical trial investigating self-assembling peptide matrix P11-4 in the prevention of artificial caries lesions.2019 | duplicate | duplicate | 2019 |
| 354 | Xu L.; Zou Y | An in Vitro Comparison of Clinpro™ XT and Duraphat Varnish for Protecting Teeth from Discoloration during Orthodontic Treatment.2023 | title/abstract | irrelevant | 2023 |
| 355 | Rana N.; Sin | A comparative evaluation of penetration depth and surface microhardness of Resin Infiltrant, CPP-ACPF and Novamin on enamel demineralization after banding: an in vitro study.2021 | title/abstract | irrelevant | 2021 |
| 356 | Lopes P.C.; | White spot lesions: diagnosis and treatment – a systematic review.2024 | title/abstract | irrelevant | 2024 |
| 357 | Al Tuma R.R. | Evaluation of three physical mixing methods of nanoparticles to orthodontic primer.2024 | title/abstract | irrelevant | 2024 |
| 358 | Ghaly Y.S.; | Effect of self-assembling peptide and other remineralizing agents on preventing initial enamel lesions around orthodontic brackets: An in vitro comparative study.2023 | duplicate | duplicate | 2023 |
| 359 | Peng S.; San | Bioinspired Anti-demineralization Enamel Coating for Orthodontics.2022 | duplicate | duplicate | 2022 |
| 360 | Montenaro M. | Influence of topical fluoride on shear bond strength of orthodontic brackets and enamel white spot lesions formation; [Influência do flúor tópico na resistência ao cisalhamento de braquetes ortodônticos e na formação de lesões de manchas brancas no esmalte].2021 | title/abstract | irrelevant | 2021 |
| 361 | Rangappa K.G | Remineralization Potential of a Combination of Chitosan with Nanohydroxyapatite and a Self-assembling Peptide with Nanohydroxyapatite: An In Vitro Study.2022 | duplicate | duplicate | 2022 |
| 362 | Satygo E.A.; | The effectiveness of professional fluoride prevention and remineralizing therapy for the initial forms of dental caries in children after orthodontic treatment.2023 | title/abstract | irrelevant | 2023 |
| 363 | Alburaiki M. | Long term remineralizing effect of casein phosphopeptide amorphous calcium phosphate in white spot lesions: A systematic review and meta-analysis.2024 | title/abstract | irrelevant | 2024 |
| 364 | Singh K.; Jh | In vitro comparative evaluation of physical and chemical properties of surface enamel after using APF and SDF with or without laser activation.2023 | title/abstract | irrelevant | 2023 |
| 365 | Aggarwal P.; | Comparison of micromorphological changes in enamel using SEM analysis after conventional and erbium, chromium:yttrium, scandium, gallium, and garnet hard-tissue laser fissurotomy: An in vitro study.2023 | duplicate | duplicate | 2023 |
| 366 | Simon L.S.; | Management of Post Orthodontic White Spot Lesions Using Resin Infiltration and CPP-ACP Materials- A Clinical Study.2022 | duplicate | duplicate | 2022 |
| 367 | Rahmanpanah | Evaluation of shear bond strength and enamel remineralizing effect of experimental orthodontic composite containing nano-hydroxyapatite: An in vitro study.2023 | duplicate | duplicate | 2023 |
| 368 | Abbassy M.A. | Characterization of a novel enamel sealer for bioactive remineralization of white spot lesions.2021 | duplicate | duplicate | 2021 |
| 369 | Fernandes G. | Antibacterial, cytotoxic and mechanical properties of a orthodontic cement with phosphate nano-sized and phosphorylated chitosan: An in vitro study.2024 | duplicate | duplicate | 2024 |
| 370 | Hu H.; Feng | Effectiveness of remineralizing agents in the prevention and reversal of orthodontically induced white spot lesions: a systematic review and network meta-analysis.2020 | duplicate | duplicate | 2020 |
| 371 | Mohanty B.; | Pediatric implants.2019 | title/abstract | irrelevant | 2019 |
| 372 | Raj S.R.; Di | Remineralization Agents in Orthodontics: Systematic Review.2023 | title/abstract | review | 2023 |
| 373 | Shaik S.N.; | A Comparative evaluation of the Shear bond strength of brackets bonded to pre-treated demineralised enamel treated with two different remineralising agents – An In-vitro study.2025 | title/abstract | irrelevant | 2025 |
| 374 | Voina Cosma | Novel Technology for Enamel Remineralization in Artificially Induced White Spot Lesions: In Vitro Study.2022 | duplicate | duplicate | 2022 |
| 375 | Nalawade V.A | Effectiveness of Various Remineralizing Agents on White Spot Lesions after Orthodontic Treatment: A Comparative Study.2021 | duplicate | duplicate | 2021 |
| 376 | Rajendran R. | Effect of remineralization agents on white spot lesions: A systematic review.2022 | title/abstract | review | 2022 |
| 377 | Jablonski-Mo | Impact of self-assembling peptides in remineralisation of artificial early enamel lesions adjacent to orthodontic brackets.2020 | duplicate | duplicate | 2020 |
| 378 | Aref N.S.; A | Casein phosphopeptide amorphous calcium phosphate and universal adhesive resin as a complementary approach for management of white spot lesions: an in-vitro study.2022 | duplicate | duplicate | 2022 |
| 379 | Verma P.; Mu | Bionic effects of nano hydroxyapatite dentifrice on demineralised surface of enamel post orthodontic debonding: in-vivo split mouth study.2021 | duplicate | duplicate | 2021 |
| 380 | Verma G.; Qu | Hydroxyapatite Nanostructured Materials for Biomedical Applications.2023 | title/abstract | irrelevant | 2023 |
| 381 | Świetlicka I | Near-surface studies of the changes to the structure and mechanical properties of human enamel under the action of fluoride varnish containing CPP–ACP compound.2020 | title/abstract | irrelevant | 2020 |
| 382 | Helal M.B.; | Comparing the remineralization potential of undemineralized dentin powder versus chicken eggshell powder on artificially induced initial enamel carious lesions: an in-vitro investigation.2024 | duplicate | duplicate | 2024 |
| 383 | Hu H.; Feng | Effectiveness of remineralising agents in prevention and treatment of orthodontically induced white spot lesions: A protocol for a systematic review incorporating network meta-analysis.2019 | title/abstract | review | 2019 |
| 384 | Loewe M.F.; | Biofilm volume and acidification within initial biofilms formed in situ on buccally and palatally exposed bracket material; [Biofilmvolumen und Azidifizierung in den ersten in situ auf bukkal und palatinal exponiertem Bracketmaterial gebildeten Biofilmen].2024 | title/abstract | irrelevant | 2024 |
| 385 | Salama R.I.; | Comparing the effect of two different remineralizing agents on shear bond strength of orthodontics brackets.2021 | title/abstract | irrelevant | 2021 |
| 386 | Setvaji N.R. | Evaluation of Remineralisation Potential of an Indigenously Developed Dentifrice – An In Vitro Study.2024 | title/abstract | irrelevant | 2024 |
| 387 | Welk A.; Rat | Effect of self-assembling peptide P11-4 on orthodontic treatment-induced carious lesions.2020 | duplicate | duplicate | 2020 |
| 388 | Agha N.N.; Q | EFFECTS OF SESAMUM INDICUM (SESAME) OIL IN REMINERALIZING OF WHITE SPOT LESIONS INDUCED AFTER BRACKET DEBONDING: AN IN VITRO STUDY.2024 | title/abstract | irrelevant | 2024 |
| 389 | Qin Q.; Yuan | A pH-sensitive, renewable invisible orthodontic aligners coating manipulates antibacterial and in situ remineralization functions to combat enamel demineralization.2024 | duplicate | duplicate | 2024 |
| 390 | Kaveh S.; Ka | Comparative Efficacy of MI Paste Plus and Fluoride Varnish Remineralizing Agents for Preventing White Spot Lesions in Patients on Fixed Orthodontic Treatment: A Clinical Trial.2022 | title/abstract | ineligible intervention | 2022 |
| 391 | Abd Elsattar | Remineralization Potential of Grape Seed Extract Versus Fluoride Mouthwash in Management of Post-Orthodontic White Spot Lesions: A 6m Randomized Clinical Trial.2023 | title/abstract | irrelevant | 2023 |
| 392 | Lin X.H.; Ya | Role and mechanism of biomimetic remineralization therapy for early enamel demineralization.2025 | title/abstract | irrelevant | 2025 |
| 393 | Warden C.; T | A novel, nitric oxide-releasing elastomeric chain for antimicrobial action: Proof of concept.2021 | duplicate | duplicate | 2021 |
| 394 | Dixit A.; Ma | Analysis of Remineralization Potential of Three Different Remineralizing Pastes on Demineralized Enamel: A Comparative Study.2021 | duplicate | duplicate | 2021 |
| 395 | Güven E.; Ed | Remineralization of post-orthodontic white spot lesions with a fluoride varnish and a self-assembling P 11 − 4 peptides: a prospective in-vivo-study.2024 | duplicate | duplicate | 2024 |
| 396 | Najjaran H.; | Effect of Diode Laser and Fluoride Varnish on Microhardness of enamel: An In Vitro Study.2024 | duplicate | duplicate | 2024 |
| 397 | Alsubhi H.; | A Comparison between Two Different Remineralizing Agents against White Spot Lesions: An in Vitro Study.2021 | title/abstract | ineligible intervention | 2021 |
| 398 | Ibrahim A.I. | Enamel conservation orthodontic paradigm via treatment with newly developed remineralizing calcium-phosphate etchant pastes.2025 | duplicate | duplicate | 2025 |
| 399 | F S.S.; Bala | Comparison of remineralizing efficacy of two novel preparation of arginine and theobromine on white spot lesion - an in vitro SEM analysis.2024 | title/abstract | irrelevant | 2024 |
| 400 | Bakry A.S.; | In-vitro comparative study for three different strategies to treat enamel demineralized white spot lesion.2025 | title/abstract | irrelevant | 2025 |
| 401 | Verma P.; Ja | Visual Assessment of Extent of White Spot Lesions in Subjects treated with Fixed Orthodontic Appliances: A Retrospective Study.2022 | title/abstract | irrelevant | 2022 |
| 402 | Anil A.; Ibr | Nano-Hydroxyapatite (nHAp) in the Remineralization of Early Dental Caries: A Scoping Review.2022 | duplicate | duplicate | 2022 |
| 403 | Shetty S.S.; | Remineralization Potential of a Novel Biomimetic Material (Self-assembling Peptide P11-4) on Early Enamel Caries: An In Vitro Study.2023 | fulltext | ineligible population | 2023 |
| 404 | Knaup T.; Ko | Effect of the caries-protective self-assembling peptide P11-4 on shear bond strength of metal brackets; [Der Einfluss des kariesprotektiven Peptids P11-4 auf den Haftverbund zwischen Metallbracket und Zahnschmelz].2021 | fulltext | ineligible population | 2021 |
| 405 | Rout T.; Pat | Remineralizing potential of Calcium Sucrose Phosphate in white spot lesions: A Systematic Review.2024 | duplicate | duplicate | 2024 |
| 406 | Gegamyan A.O | Evaluation of enamel remineralization rate by quantitative light-induced fluorescence; [Оценка скорости реминерализации эмали при помощи количественной светоиндуцированной флуоресценции].2021 | title/abstract | ineligible intervention | 2021 |
| 407 | Ciribè M.; C | Efficacy of F-ACP-Containing Dental Mousse in the Remineralization of White Spot Lesions after Fixed Orthodontic Therapy: A Randomized Clinical Trial.2024 | title/abstract | irrelevant | 2024 |
| 408 | Mollabashi V | Evaluation of Nano TiO2 Modified Orthodontic Composite Effects on S. mutans Population and Enamel Demineralization in Fixed Orthodontic Patients; a Split Mouth Randomized Controlled Clinical Trial.2023 | title/abstract | irrelevant | 2023 |
| 409 | Nookala H.; | Microbial Biofilm Inhibition in Dental White Spot Lesions using Crustin Derived Antimicrobial Peptide Crustin (CAMP) and Bio-assisted Sida Acuta Mediated Titanium Nanoparticles (SA_NP).2024 | title/abstract | irrelevant | 2024 |
| 410 | Wan Hassan W | A 12-months randomized clinical trial comparing fluoride-based remineralising protocols on post-orthodontic initial caries lesions.2025 | duplicate | duplicate | 2025 |
| 411 | Hong S.-C.; | Micro-computed tomographic evaluation of the effect of fluoride agents on white spot lesions: An in vitro study.2022 | duplicate | duplicate | 2022 |
| 412 | Sain S.; Heg | Remineralization of Enamel Using Topical Agents among Patients with Orthodontic Brackets: In Vivo and In Vitro Randomized Control Trial.2024 | duplicate | duplicate | 2024 |
| 413 | Leite K.L.F. | In-vitro effect of a single application of CPP-ACP pastes and different fluoridated solutions on the prevention of dental caries around orthodontic brackets.2023 | title/abstract | ineligible intervention | 2023 |
| 414 | Lena Sezici | Comparative evaluation of fluoride varnishes, self-assembling peptide-based remineralization agent, and enamel matrix protein derivative on artificial enamel remineralization in vitro.2021 | duplicate | duplicate | 2021 |
| 415 | Heravi F.; B | Evaluation of Microleakage of Orthodontic Bands Cemented With CPP-ACP-Modified Glass Ionomer Cement.2019 | duplicate | duplicate | 2019 |
| 416 | Aboulnaga M. | Evaluation of Remineralization Potential of Remin Pro Forte vs Remin Pro on White Spot Lesions: A Randomized Clinical Trial.2022 | title/abstract | irrelevant | 2022 |
| 417 | Bandeira Lop | Molar-incisor hypomineralization: an umbrella review.2021 | duplicate | duplicate | 2021 |
| 418 | Abuzenada B. | Bionanocomposites in caries prevention and treatment: A systematic review.2022 | duplicate | duplicate | 2022 |
| 419 | Ghadirian H. | The effect of remineralizing agents with/without CO2 laser irradiation on structural and mechanical properties of enamel and its shear bond strength to orthodontic brackets.2020 | title/abstract | irrelevant | 2020 |
| 420 | Subramanian | AN IN VITRO STUDY ON THE EFFICACY OF FOUR REMINERALIZING AGENTS.2022 | title/abstract | ineligible intervention | 2022 |
| 421 | Hamed S.; Ab | Fluoride-amorphous calcium phosphate and biomimetic nano-hydroxyapatite for enamel remineralization: An in-vitro study of surface microhardness and composition.2024 | duplicate | duplicate | 2024 |
| 422 | Butera A.; P | Biomimetic hydroxyapatite paste for molar–incisor hypomineralization: A randomized clinical trial.2023 | title/abstract | irrelevant | 2023 |
| 423 | Abo-Elmahase | Evaluation of the remineralizing effect of the chicken eggshell paste after removal of the fixed orthodontic appliance: An in vitro study.2024 | title/abstract | irrelevant | 2024 |
| 424 | Moraes S.M.; | Effectiveness of Fluoride Varnishes for White Spot Lesion Prevention and Remineralization during Orthodontic Treatment: A Randomized Controlled Trial.2024 | duplicate | duplicate | 2024 |
| 425 | Hamdi K.; El | Remineralization and anti-demineralization effect of orthodontic adhesives on enamel surrounding orthodontic brackets: a systematic review of in vitro studies.2024 | title/abstract | irrelevant | 2024 |
| 426 | Wierichs R.J | Short-Term efficacy of caries resin infiltration during treatment with orthodontic fixed appliances. A randomized controlled trial.2023 | duplicate | duplicate | 2023 |
| 427 | Gohar R.A.A. | Evaluation of the remineralizing effect of biomimetic self-assembling peptides in post-orthodontic white spot lesions compared to fluoride-based delivery systems: randomized controlled trial.2023 | duplicate | duplicate | 2023 |
| 428 | Scribante A. | Biomimetic Effect of Nano-Hydroxyapatite in Demineralized Enamel before Orthodontic Bonding of Brackets and Attachments: Visual, Adhesion Strength, and Hardness in in Vitro Tests.2020 | duplicate | duplicate | 2020 |
| 429 | Reddy V.S.; | A comparative evaluation of human enamel remineralization ability of biomimetic nacre against casein phosphopeptide‑amorphous calcium phosphate: An in vitro study.2024 | title/abstract | irrelevant | 2024 |
| 430 | Peng S.; Gua | Efficacy of peptide-based enamel coatings in the prevention of demineralization using fixed orthodontic brackets in a rat model.2024 | duplicate | duplicate | 2024 |
| 431 | Enan E.; Taw | Remineralization Potential and Shear Bond Strength of Surface Treated Hypomineralized Enamel in Bonding of Orthodontic Brackets: An In Vitro Study.2021 | duplicate | duplicate | 2021 |
| 432 | Pushpalatha | Nanohydroxyapatite in dentistry: A comprehensive review.2023 | title/abstract | irrelevant | 2023 |
| 433 | Pavethynath | Evaluation of Prevention of Initial Enamel Lesions around Orthodontic Brackets by Using Different Remineralizing Agents: An Original Research.2024 | duplicate | duplicate | 2024 |
| 434 | Bhat D.V.; A | Evaluation of Remineralizing Potential of CPP-ACP, CPP-ACP + F and β TCP + F and Their Effect on Microhardness of Enamel Using Vickers Microhardness Test: An In Vitro Study.2022 | title/abstract | irrelevant | 2022 |
| 435 | Butera A.; C | The effect of seven preventive treatments containing microrepair on the shear bond strengths of orthodontic brackets: An in vitro study.2020 | title/abstract | irrelevant | 2020 |
| 436 | YU J.; QIN D | THE EFFECTS OF PREVENTIVE INTERVENTIONS FOR ORTHODONTICALLY INDUCED WHITE SPOT LESIONS: AN UMBRELLA REVIEW AND EVIDENCE MAPPING.2025 | duplicate | duplicate | 2025 |
| 437 | Linjawi A.I. | Sealants and white spot lesions in orthodontics: A review.2020 | duplicate | duplicate | 2020 |
| 438 | Danisman H.; | Evaluation of the efficacy of P11-4 and CCP-ACPF in the prevention and treatment of white spot lesions: a multi-technique approach.2025 | duplicate | duplicate | 2025 |
| 439 | Shakir M.; M | Innovation of nano-hydrogels loaded with amelogenin peptide and hydroxyapatite nano-particles for remineralisation of artificially induced white spot lesions.2024 | duplicate | duplicate | 2024 |
| 440 | Chen Y.; Che | Bio-inspired nanocomposite coatings on orthodontic archwires with corrosion resistant and antibacterial properties.2023 | title/abstract | irrelevant | 2023 |
| 441 | Ludovichetti | Prevention of White Spot Lesions Induced by Fixed Orthodontic Therapy: A Literature Review.2025 | duplicate | duplicate | 2025 |
| 442 | Yu C.; Shen | Rapid detection of enamel demineralization based on supramolecular fluorescent probes in vitro.2024 | duplicate | duplicate | 2024 |
| 443 | Bian C.; Guo | New generation of orthodontic devices and materials with bioactive capacities to improve enamel demineralization.2024 | title/abstract | irrelevant | 2024 |
| 444 | Jablonski-Mo | Impact of self-assembling peptides in remineralization of early enamel lesions adjacent to orthodontic brackets detected by bioluminescence measurements; [Einfluss von Self-assembling Peptiden auf die Remineralisation initialkari ser L sionen am Bracketrand nachgewiesen mittels Biolumineszenzverfahren].2020 | title/abstract | irrelevant | 2020 |
| 445 | Mollabashi V | Remineralization Effect of Bioactive glass Toothpaste Versus Sodium Fluoride Toothpaste on White Spot Lesions Around Orthodontic Brackets: An In Vitro Study.2022 | title/abstract | irrelevant | 2022 |
| 446 | Ghadirian H. | Efficacy of Enamel Remineralizing Agents in Prevention of Dental Caries Around and Beneath Orthodontic Brackets.2022 | title/abstract | irrelevant | 2022 |
| 447 | Bakhsh T.; A | Effect of bioglass on artificially induced enamel lesion around orthodontic brackets: OCT study.2018 | title/abstract | irrelevant | 2018 |
| 448 | Purdell-Lewi | Microhardness and densitometric measurements of the effect of 4% snf2 solution on artificial white spot lesions.1976 | title/abstract | irrelevant | 1976 |
| 449 | Dai Z.; Liu | Effects of Fluoride and Calcium Phosphate Materials on Remineralization of Mild and Severe White Spot Lesions.2019 | duplicate | duplicate | 2019 |
| 450 | Giannetti L. | Superficial infiltration to treat white hypomineralized defects of enamel: Clinical trial with 12-month follow-up.2018 | title/abstract | irrelevant | 2018 |
| 451 | Shirazi M.; | Effect of addition of bioactive glass to resin modified glass ionomer cement on enamel demineralization under orthodontic brackets.2019 | duplicate | duplicate | 2019 |
| 452 | Baroni C.; M | A SEM and non-contact surface white light profilometry in vivo study of the effect of a crème containing CPP-ACP and fluoride on young etched enamel.2014 | title/abstract | irrelevant | 2014 |
| 453 | Splieth C.H. | Caries-preventive and remineralizing effect of fluoride gel in orthodontic patients after 2 years.2012 | duplicate | duplicate | 2012 |
| 454 | Chen H.; Liu | Effect of remineralizing agents on white spot lesions after orthodontic treatment: A systematic review.2013 | duplicate | duplicate | 2013 |
| 455 | Butera A.; M | The effect of six different preventive treatments on the shear bond strengths of orthodontic brackets: An in vitro study.2019 | title/abstract | irrelevant | 2019 |
| 456 | Fernández-Fe | Enamel remineralization therapies for treating postorthodontic white-spot lesions: A systematic review.2018 | duplicate | duplicate | 2018 |
| 457 | Raj B.J.R.; | Remineralising agents in dentistry.2016 | title/abstract | irrelevant | 2016 |
| 458 | Brown M.L.; | Ion release from a novel orthodontic resin bonding agent for the reduction and/or prevention of white spot lesions An in vitro study.2011 | title/abstract | irrelevant | 2011 |
| 459 | Ma Y.; Zhang | Novel multifunctional dental cement to prevent enamel demineralization near orthodontic brackets.2017 | fulltext | ineligible intervention | 2017 |
| 460 | Bansal K.; B | Remineralizing efficacy of Calcarea Fluorica tablets on the artificial carious enamel lesions using scanning electron microscope and surface microhardness testing: In vivostudy.2014 | title/abstract | irrelevant | 2014 |
| 461 | Baeshen H.A. | Effect of fluoridated chewing sticks (Miswaks) on white spot lesions in postorthodontic patients.2011 | duplicate | duplicate | 2011 |
| 462 | Casals E.; B | Anticaries potential of commercial dentifrices as determined by fluoridation and remineralization efficiency.2007 | title/abstract | irrelevant | 2007 |
| 463 | Yadav P.; De | A comparative quantitative & qualitative assessment in orthodontic treatment of white spot lesion treated with 3 different commercially available materials - In vitro study.2019 | title/abstract | irrelevant | 2019 |
| 464 | Rubel M.; Pr | Effect of grape seed extract on remineralization of artificial caries: An in-vitro study.2016 | title/abstract | irrelevant | 2016 |
| 465 | Bakry A.S.; | A novel fluoride containing bioactive glass paste is capable of re-mineralizing early caries lesions.2018 | duplicate | duplicate | 2018 |
| 466 | Yassaei S.; | Efficacy of erbium-doped yttrium aluminum garnet laser with casein phosphopeptide amorphous calcium phosphate with and without fluoride for remineralization of white spot lesions around orthodontic brackets.2018 | title/abstract | ineligible intervention | 2018 |
| 467 | Patil N.; Ja | In vitro caries-preventive effect of fluoridated orthodontic resins against cariogenic challenge stimulation.2012 | duplicate | duplicate | 2012 |
| 468 | Suetenkov D. | Photo activated disinfection efficiency of low-intensity laser and comprehensive prevention of caries and gingivitis in adolescents using bracket system.2015 | title/abstract | irrelevant | 2015 |
| 469 | Burbank B.D. | Ion release, fluoride charge of and adhesion of an orthodontic cement paste containing microcapsules.2016 | title/abstract | irrelevant | 2016 |
| 470 | Linton J.L. | Quantitative measurements of remineralization of incipient caries..1996 | title/abstract | irrelevant | 1996 |
| 471 | Bril' E.A.; | Primary prevention of the main dental diseases in children at different stages of orthodontic treatment with bracket systems; [Первичная профилактика основных стоматологических заболеваний у детей на этапах лечения брекет-системой].2019 | title/abstract | irrelevant | 2019 |
| 472 | Elumalai M.; | Remineralizing effect of zinc reinforced synthetic nano-hydroxyapatite on caries-like lesion in human permanent teeth - An in vitro study.2018 | title/abstract | irrelevant | 2018 |
| 473 | Kohda N.; Ii | Inhibition of enamel demineralization and bond-strength properties of bioactive glass containing 4-META/MMA-TBB-based resin adhesive.2015 | duplicate | duplicate | 2015 |
| 474 | Yeoh E.S.; L | Clinical evidence in the treatment of white spot lesions following fixed orthodontic therapy: A meta-analysis.2018 | title/abstract | irrelevant | 2018 |
| 475 | Uy E.; Ekamb | Remineralization potential of calcium and phosphate-based agents and their effects on bonding of orthodontic brackets.2019 | duplicate | duplicate | 2019 |
| 476 | Gurunathan D | Prevention of white spot lesion in orthodontic patients using casein phosphopeptide-stabilized amorphous calcium phosphate -a systematic review.2015 | title/abstract | review | 2015 |
| 477 | Ben Mohimd H | Enamel protection after stripping procedures: An in vivo study; [La protection de l’émail après procédures de stripping: étude in vivo].2019 | title/abstract | irrelevant | 2019 |
| 478 | Jablonski-Mo | Efficacy of the self-assembling peptide P11-4 in constructing a remineralization scaffold on artificially-induced enamel lesions on smooth surfaces; [Wirksamkeit des Self-assembling Peptid P11-4 zur Bildung einer Remineralisationsmatrix auf artifiziell erzeugten Schmelzläsionen an Glattflächen].2014 | duplicate | duplicate | 2014 |
| 479 | Somasundaram | Protective potential of casein phosphopeptide amorphous calcium phosphate containing paste on enamel surfaces.2013 | duplicate | duplicate | 2013 |
| 480 | Heravi F.; B | An in vitro study on the retentive strength of orthodontic bands cemented with CPP-ACP-containing GIC.2016 | title/abstract | irrelevant | 2016 |
| 481 | Samuel S.R.; | Effect of ozone to remineralize initial enamel caries: in situ study.2016 | duplicate | duplicate | 2016 |
| 482 | Wang J.-X.; | Clinical evaluation of remineralization potential of casein phosphopeptide amorphous calcium phosphate nanocomplexes for enamel decalcification in orthodontics.2012 | duplicate | duplicate | 2012 |
| 483 | Langhorst S. | In vitro remineralization of enamel by polymeric amorphous calcium phosphate composite: Quantitative microradiographic study.2009 | duplicate | duplicate | 2009 |
| 484 | Singh S.; Si | Effects of various remineralizing agents on the outcome of post-orthodontic white spot lesions (WSLs): a clinical trial.2016 | title/abstract | irrelevant | 2016 |
| 485 | Iijima M.; I | Effects of the addition of fluoride to a 4-META/MMA-TBB-based resin adhesive on fluoride release, acid resistance of enamel and shear bond strength in vitro.2013 | title/abstract | irrelevant | 2013 |
| 486 | Chin, MYH; S | Fluoride release and recharge potential of remineralizing orthodontic adhesive systems.2019 | duplicate | duplicate | 2019 |
| 487 | Beerens M.W. | Long-term remineralizing effect of MI Paste Plus on regression of early caries after orthodontic fixed appliance treatment: A 12-month follow-up randomized controlled trial.2018 | title/abstract | irrelevant | 2018 |
| 488 | Majithia U.; | Comparative evaluation of application of different fluoride varnishes on artificial early enamel lesion: An in vitro study.2016 | duplicate | duplicate | 2016 |
| 489 | Catros S.; P | Collagen fibrils of human acellular extrinsic fiber cementum.2008 | duplicate | duplicate | 2008 |
| 490 | Bergstrand F | A review on prevention and treatment of post-orthodontic white spot lesions - evidence-based methods and emerging technologies.2011 | duplicate | duplicate | 2011 |
| 491 | Indrapriyadh | Remineralizing potential of CPP-ACP in white spot lesions-A systematic review.2018 | title/abstract | review | 2018 |
| 492 | Altmann A.S. | In vitro antibacterial and remineralizing effect of adhesive containing triazine and niobium pentoxide phosphate inverted glass.2017 | duplicate | duplicate | 2017 |
| 493 | Raphael S.; | Casein phosphopeptide-amorphous calcium phosphate products in caries prevention.2016 | title/abstract | irrelevant | 2016 |
| 494 | Garry A.P.; | A randomised controlled trial to investigate the remineralising potential of Tooth Mousse™ in orthodontic patients.2017 | title/abstract | irrelevant | 2017 |
| 495 | Walsh L.J.; | Prevention and caries risk management in teenage and orthodontic patients.2019 | title/abstract | irrelevant | 2019 |
| 496 | Korkut B.; K | Clinical assessment of demineralization and remineralization surrounding orthodontic brackets with FluoreCam.2017 | title/abstract | ineligible intervention | 2017 |
| 497 | Pretty I.A.; | The in vitro detection of early enamel de- and re-mineralization adjacent to bonded orthodontic cleats using quantitative light-induced fluorescence.2003 | duplicate | duplicate | 2003 |
| 498 | Chin M.Y.H.; | Fluoride release and cariostatic potential of orthodontic adhesives with and without daily fluoride rinsing.2009 | duplicate | duplicate | 2009 |
| 499 | Bhongsatiern | Adjunctive use of fluoride rinsing and brush-on gel increased incipient caries-like lesion remineralization compared with fluoride toothpaste alone in situ.2019 | duplicate | duplicate | 2019 |
| 500 | Melo M.A.S.; | Nanotechnology-based restorative materials for dental caries management.2013 | title/abstract | irrelevant | 2013 |
| 501 | Songsiriprad | Sodium fluoride mouthrinse used twice daily increased incipient caries lesion remineralization in an in situ model.2014 | duplicate | duplicate | 2014 |
| 502 | Malterud M.I | Minimally invasive restorative dentistry: a biomimetic approach..2006 | title/abstract | irrelevant | 2006 |
| 503 | Bakhsh T.A.; | Novel evaluation and treatment techniques for white spot lesions. An in vitro study.2017 | title/abstract | irrelevant | 2017 |
| 504 | Munjal D.; G | Assessment of white spot lesions and in-vivo evaluation of the effect of CPP-ACP on white spot lesions in permanent molars of children.2016 | duplicate | duplicate | 2016 |
| 505 | Andersson A. | Effect of a dental cream containing amorphous calcium phosphate complexes on white spot lesion regression assessed by laser fluorescence.2007 | title/abstract | ineligible intervention | 2007 |
| 506 | Kleber C.J.; | Treatment of Orthodontic White Spot Lesions with a Remineralizing Dentifrice Applied by Toothbrushing or Mouth Trays.1999 | duplicate | duplicate | 1999 |
| 507 | Ferreira C.J | Antibacterial and remineralizing fillers in experimental orthodontic adhesives.2019 | duplicate | duplicate | 2019 |
| 508 | Kalha A.S. | Lack of reliable evidence of the effectiveness of remineralising agents for the treatment of post orthodontic white spot lesions.2013 | title/abstract | irrelevant | 2013 |
| 509 | Mahmood H.T. | Application of new biomedical materials in orthodontic appliances.2019 | duplicate | duplicate | 2019 |
| 510 | Manfred L.; | A novel biomimetic orthodontic bonding agent helps prevent white spot lesions adjacent to brackets.2013 | duplicate | duplicate | 2013 |
| 511 | Passalini P. | Mechanical properties of one and twostep fluoridated orthodontic resins submitted to different pH cycling regimes.2010 | title/abstract | irrelevant | 2010 |
| 512 | Passalini P. | Preventive effect of fluoridated orthodontic resins subjected to high cariogenic challenges.2010 | duplicate | duplicate | 2010 |
| 513 | Karad A.; Dh | White spot lesions in orthodontic patients: An expert opinion.2019 | title/abstract | expert opinion | 2019 |
| 514 | Mohanty P.; | An in vitro evaluation of remineralization potential of novamin® on artificial enamel sub-surface lesions around orthodontic brackets using energy dispersive x-ray analysis (EDX).2014 | title/abstract | irrelevant | 2014 |
| 515 | Mayne R.J.; | In-vitro study of the effect of casein phosphopeptide amorphous calcium fluoride phosphate on iatrogenic damage to enamel during orthodontic adhesive removal.2011 | duplicate | duplicate | 2011 |
| 516 | Ogihara S.; | Efficacy of forced eruption/enamel matrix derivative with freeze-dried bone allograft or with demineralized freeze-dried bone allograft in infrabony defects: A randomized trial.2015 | duplicate | duplicate | 2015 |
| 517 | Mohapatra S. | Assessment of microhardness of enamel carious like lesions after treatment with nova min, bio min and remin pro containing toothpastes: An in vitro study.2019 | title/abstract | irrelevant | 2019 |
| 518 | Bailey D.L.; | Regression of post-orthodontic lesions by a remineralizing cream.2009 | duplicate | duplicate | 2009 |
| 519 | Liu Y.; Zhan | Antibacterial and remineralizing orthodontic adhesive containing quaternary ammonium resin monomer and amorphous calcium phosphate nanoparticles.2018 | duplicate | duplicate | 2018 |
| 520 | Brignardello | There are probably no differences in arrest of white-spot lesions and plaque composition between remineralizing and nonfluoridated toothpastes.2018 | duplicate | duplicate | 2018 |
| 521 | Øgaard B.; R | Orthodontic appliances and enamel demineralization Part 2. Prevention and treatment of lesions.1988 | title/abstract | irrelevant | 1988 |
| 522 | Bansal K.; G | In vivo remineralization of artificial enamel carious lesions using a mineral-enriched mouthrinse and a fluoride dentifrice: A polarized light microscopic comparative evaluation.2010 | title/abstract | irrelevant | 2010 |
| 523 | Zero D.T. | Application of Clinical Models in Remineralization Research.1999 | duplicate | duplicate | 1999 |
| 524 | Heravi F.; A | Effectiveness of MI Paste Plus and Remin Pro on remineralization and color improvement of postorthodontic white spot lesions.2018 | title/abstract | irrelevant | 2018 |
| 525 | Sabatini, S; | Protocols and Technologies Used by Italian Dental Professionals to Maintain Good Oral Health in Orthodontic Patients Before, During and After Treatment: A Survey Study.2025 | title/abstract | irrelevant | 2025 |
| 526 | Wang, JX; Ya | Clinical evaluation of remineralization potential of casein phosphopeptide amorphous calcium phosphate nanocomplexes for enamel decalcification in orthodontics.2012 | duplicate | duplicate | 2012 |
| 527 | Munjal, D; G | Assessment of White Spot Lesions and In-Vivo Evaluation of the Effect of CPP-ACP on White Spot Lesions in Permanent Molars of Children.2016 | title/abstract | ineligible intervention | 2016 |
| 528 | Karad, A; Dh | White spot lesions in orthodontic patients: An expert opinion.2019 | duplicate | duplicate | 2019 |
| 529 | Bailey, DL; | Regression of Post-orthodontic Lesions by a Remineralizing Cream.2009 | title/abstract | ineligible intervention | 2009 |
| 530 | Kleber, CJ; | Treatment of orthodontic white spot lesions with a remineralizing dentifrice applied by toothbrushing or mouth trays.1999 | duplicate | duplicate | 1999 |
| 531 | Samuel, SR; | Effect of ozone to remineralize initial enamel caries: in situ study.2016 | duplicate | duplicate | 2016 |
| 532 | Thorat, SU; | Evaluating the Remineralizing Effects of Calcium Sucrose Phosphate and Casein Phosphopeptide-Amorphous Calcium Phosphate Toothpastes on Artificial Carious Lesions Using Micro-computed Tomography: An In Vitro Investigation.2024 | title/abstract | ineligible intervention | 2024 |
| 533 | Altmann, ASP | In vitro antibacterial and remineralizing effect of adhesive containing triazine and niobium pentoxide phosphate inverted glass.2017 | duplicate | duplicate | 2017 |
| 534 | Fernandes, G | Antibacterial, cytotoxic and mechanical properties of a orthodontic cement with phosphate nano-sized and phosphorylated chitosan: An in vitro study.2024 | title/abstract | irrelevant | 2024 |
| 535 | Baeshen, HA; | Effect of fluoridated chewing sticks (Miswaks) on white spot lesions in postorthodontic patients.2011 | title/abstract | irrelevant | 2011 |
| 536 | Yacout, YM; | Shear bond strength of metallic brackets bonded to enamel pretreated with CPP-ACP: a systematic review and meta-analysis of in vitro studies.2023 | title/abstract | irrelevant | 2023 |
| 537 | Vas, NV; Jai | Self-Assembling Peptide P11-4 For Management Of White Spot Lesions In Subjects Undergoing Orthodontic Treatment. - A Structured Review.2024 | title/abstract | irrelevant | 2024 |
| 538 | Pavethynath, | Evaluation of Prevention of Initial Enamel Lesions around Orthodontic Brackets by Using Different Remineralizing Agents: An Original Research.2024 | duplicate | duplicate | 2024 |
| 539 | Linton, JL | Quantitative measurements of remineralization of incipient caries.1996 | duplicate | duplicate | 1996 |
| 540 | Welk, A; Rat | Effect of self-assembling peptide P11-4 on orthodontic treatment-induced carious lesions.2020 | duplicate | duplicate | 2020 |
| 541 | OGAARD, B; R | ORTHODONTIC APPLIANCES AND ENAMEL DEMINERALIZATION .2. PREVENTION AND TREATMENT OF LESIONS.1988 | title/abstract | irrelevant | 1988 |
| 542 | Ludovichetti | Prevention of White Spot Lesions Induced by Fixed Orthodontic Therapy: A Literature Review.2025 | duplicate | duplicate | 2025 |
| 543 | Bakry, AS; A | A Novel Fluoride Containing Bioactive Glass Paste is Capable of Re-Mineralizing Early Caries Lesions.2018 | fulltext | irrelevant | 2018 |
| 544 | Ghaly, YS; E | Effect of self-assembling peptide and other remineralizing agents on preventing initial enamel lesions around orthodontic brackets: An in vitro comparative study.2023 | duplicate | duplicate | 2023 |
| 545 | Helal, MB; S | Comparing the remineralization potential of undemineralized dentin powder versus chicken eggshell powder on artificially induced initial enamel carious lesions: an invitro investigation.2024 | title/abstract | irrelevant | 2024 |
| 546 | Al-Blaihed, | White Spot Lesions in Fixed Orthodontics: A Literature Review on Etiology, Prevention, and Treatment.2024 | duplicate | duplicate | 2024 |
| 547 | Fernández-Fe | Enamel remineralization therapies for treating postorthodontic white-spot lesions A systematic review.2018 | title/abstract | irrelevant | 2018 |
| 548 | Jablonski-Mo | Impact of self-assembling peptides in remineralisation of artificial early enamel lesions adjacent to orthodontic brackets.2020 | duplicate | duplicate | 2020 |
| 549 | Gohar, RAAG; | Evaluation of the remineralizing effect of biomimetic self-assembling peptides in post-orthodontic white spot lesions compared to fluoride-based delivery systems: randomized controlled trial.2023 | duplicate | duplicate | 2023 |
| 550 | Senthilkumar | Assessment of the Additive Effect of Remineralizing Agents in Combination With Fluoride Releasing Adhesives in the Prevention of Enamel Decalcification in Orthodontic Patients: An In Vitro Study.2024 | duplicate | duplicate | 2024 |
| 551 | Mollabashi, | Evaluation of Nano TiO2 Modified Orthodontic Composite Effects on S. mutans Population and Enamel Demineralization in Fixed Orthodontic Patients; a Split Mouth Randomized Controlled Clinical Trial.2023 | duplicate | duplicate | 2023 |
| 552 | Yu, CR; Shen | Rapid detection of enamel demineralization based on supramolecular fluorescent probes in vitro.2024 | title/abstract | irrelevant | 2024 |
| 553 | Bian, C; Guo | New generation of orthodontic devices and materials with bioactive capacities to improve enamel demineralization.2024 | duplicate | duplicate | 2024 |
| 554 | Ma, YS; Zhan | Novel multifunctional dental cement to prevent enamel demineralization near orthodontic brackets.2017 | duplicate | duplicate | 2017 |
| 555 | Knaup, T; Ko | Effect of the caries-protective self-assembling peptide P11-4 on shear bond strength of metal brackets.2021 | duplicate | duplicate | 2021 |
| 556 | Uy, E; Ekamb | Remineralization Potential of Calcium and Phosphate-based Agents and Their Effects on Bonding of Orthodontic Brackets.2019 | duplicate | duplicate | 2019 |
| 557 | Peng, S; San | Bioinspired Anti-demineralization Enamel Coating for Orthodontics.2022 | title/abstract | irrelevant | 2022 |
| 558 | Macovei, G; | ASSESSMENT OF BACTERIAL BIOFILM ON PATIENTS WITH ORTHODONTIC FIXED APPLIANCES FOLLOWING NON OPERATIVE/PREVENTIVE TREATMENTS.2016 | title/abstract | irrelevant | 2016 |
| 559 | Anil, A; Ibr | Nano-Hydroxyapatite (nHAp) in the Remineralization of Early Dental Caries: A Scoping Review.2022 | duplicate | duplicate | 2022 |
| 560 | Mahmood, HT; | Application of New Biomedical Materials in Orthodontic Appliances.2019 | duplicate | duplicate | 2019 |
| 561 | Jablonski-Mo | Efficacy of the self-assembling peptide P11-4 in constructing a remineralization scaffold on artificially-induced enamel lesions on smooth surfaces.2014 | duplicate | duplicate | 2014 |
| 562 | Seifi, M; Es | Investigation of mechanical properties, remineralization, antibacterial effect, and cellular toxicity of composite orthodontic adhesive combined with silver-containing nanostructured bioactive glass.2024 | duplicate | duplicate | 2024 |
| 563 | Chen, H; Liu | Effect of remineralizing agents on white spot lesions after orthodontic treatment: A systematic review.2013 | duplicate | duplicate | 2013 |
| 564 | Hu, HM; Feng | Effectiveness of remineralizing agents in the prevention and reversal of orthodontically induced white spot lesions: a systematic review and network meta-analysis.2020 | duplicate | duplicate | 2020 |
| 565 | Bakhsh, TA; | Novel evaluation and treatment techniques for white spot lesions. An in vitro study.2017 | duplicate | duplicate | 2017 |
| 566 | Warden, C; T | A novel, nitric oxide-releasing elastomeric chain for antimicrobial action: proof of concept.2021 | title/abstract | irrelevant | 2021 |
| 567 | Singh, S; Si | Effects of various remineralizing agents on the outcome of post-orthodontic white spot lesions (WSLs): a clinical trial.2016 | fulltext | ineligible intervention | 2016 |
| 568 | Hamdi, K; El | Remineralization and anti-demineralization effect of orthodontic adhesives on enamel surrounding orthodontic brackets: a systematic review of in vitro studies.2024 | duplicate | duplicate | 2024 |
| 569 | Bakry, AS; A | In-vitro comparative study for three different strategies to treat enamel demineralized white spot lesion.2025 | duplicate | duplicate | 2025 |
| 570 | Danisman, H; | Evaluation of the efficacy of P11-4 and CCP-ACPF in the prevention and treatment of white spot lesions: a multi-technique approach.2025 | title/abstract | ineligible intervention | 2025 |
| 571 | Abuzenada, B | Bionanocomposites in Caries Prevention and Treatment: A Systematic Review.2022 | duplicate | duplicate | 2022 |
| 572 | Manfred, L; | A novel biomimetic orthodontic bonding agent helps prevent white spot lesions adjacent to brackets.2013 | duplicate | duplicate | 2013 |
| 573 | Moraes, SM; | Effectiveness of Fluoride Varnishes for White Spot Lesion Prevention and Remineralization during Orthodontic Treatment: A Randomized Controlled Trial.2024 | title/abstract | ineligible intervention | 2024 |
| 574 | Ghadirian, H | The Effect of Remineralizing Agents With/Without CO2 Laser Irradiation on Structural and Mechanical Properties of Enamel and its Shear Bond Strength to Orthodontic Brackets.2020 | duplicate | duplicate | 2020 |
| 575 | Rout, T; Pat | Remineralizing potential of Calcium Sucrose Phosphate in white spot lesions: A Systematic Review.2024 | duplicate | duplicate | 2024 |
| 576 | Moslehitabar | Efficacy of an Experimental CPP-ACP and Fluoride Toothpaste in Prevention of Enamel Demineralization: An In Vitro Study on Bovine Enamel.2025 | title/abstract | animal study | 2025 |
| 577 | Alsubhi, H; | A Comparison between Two Different Remineralizing Agents against White Spot Lesions: An In Vitro Study.2021 | duplicate | duplicate | 2021 |
| 578 | Golland, L; | The Potential of Self-assembling Peptides for Enhancement of In Vitro Remineralisation of White Spot Lesions as Measured by Quantitative Laser Fluorescence.2017 | fulltext | ineligible population | 2017 |
| 579 | Gueven, E; E | Remineralization of post-orthodontic white spot lesions with a fluoride varnish and a self-assembling P 11-4 peptides: a prospective in-vivo-study.2024 | duplicate | duplicate | 2024 |
| 580 | Liu, Y; Zhan | Antibacterial and remineralizing orthodontic adhesive containing quaternary ammonium resin monomer and amorphous calcium phosphate nanoparticles.2018 | duplicate | duplicate | 2018 |
| 581 | Savio, FS; B | Comparison of remineralizing efficacy of two novel preparation of arginine and theobromine on white spot lesion - an in vitro SEM analysis.2024 | duplicate | duplicate | 2024 |
| 582 | Lopes, PC; C | White spot lesions: diagnosis and treatment - a systematic review.2024 | duplicate | duplicate | 2024 |
| 583 | Kawamura, N; | Wear characteristics and inhibition of enamel demineralization by resin-based coating materials.2017 | title/abstract | irrelevant | 2017 |
| 584 | Chin, MYH; S | Fluoride release and cariostatic potential of orthodontic adhesives with and without daily fluoride rinsing.2009 | title/abstract | irrelevant | 2009 |
| 585 | Bakhsh, T; A | Effect of bioglass on artificially induced enamel lesion around orthodontic brackets: OCT study.2018 | duplicate | duplicate | 2018 |
| 586 | Kohda, N; Ii | Inhibition of enamel demineralization and bond-strength properties of bioactive glass containing 4-META/MMA-TBB-based resin adhesive.2015 | title/abstract | irrelevant | 2015 |
| 587 | Xie, ZX; Yu, | Comparison of therapies of white spot lesions: a systematic review and network meta-analysis.2023 | duplicate | duplicate | 2023 |
| 588 | Jablonski-Mo | Randomised in situ clinical trial investigating self-assembling peptide matrix P11-4 in the prevention of artificial caries lesions.2019 | duplicate | duplicate | 2019 |
| 589 | Nuñez-Solano | Effects of Three Remineralizing Agents in the Shear Bond Strength of Orthodontic Brackets.2022 | title/abstract | irrelevant | 2022 |
| 590 | Lu, XW; Qu, | Applications of photothermally mediated nanohybrids for white spot lesions in orthodontics.2023 | duplicate | duplicate | 2023 |
| 591 | Enan, E; Taw | Remineralization Potential and Shear Bond Strength of Surface Treated Hypomineralized Enamel in Bonding of Orthodontic Brackets: An In Vitro Study.2021 | title/abstract | irrelevant | 2021 |
| 592 | Chen, YM; Ch | Bio-inspired nanocomposite coatings on orthodontic archwires with corrosion resistant and antibacterial properties.2023 | duplicate | duplicate | 2023 |
| 593 | Aref, NS; Al | Surface topography and spectrophotometric assessment of white spot lesions restored with nano-hydroxyapatite-containing universal adhesive resin: an in-vitro study.2023 | duplicate | duplicate | 2023 |
| 594 | Kondelova, P | Efficacy of P11-4 for the treatment of initial buccal caries: a randomized clinical trial.2020 | fulltext | ineligible population | 2020 |
| 595 | Dashper, SG; | CPP-ACP Promotes SnF2 Efficacy in a Polymicrobial Caries Model.2019 | title/abstract | irrelevant | 2019 |
| 596 | Verma, P; Pa | Bionic effects of nano hydroxyapatite dentifrice on demineralised surface of enamel post orthodontic debonding: in-vivo split mouth study.2021 | duplicate | duplicate | 2021 |
| 597 | Cosma, LLV; | Novel Technology for Enamel Remineralization in Artificially Induced White Spot Lesions: In Vitro Study.2022 | title/abstract | irrelevant | 2022 |
| 598 | Aref, NS; Al | Casein phosphopeptide amorphous calcium phosphate and universal adhesive resin as a complementary approach for management of white spot lesions: an in-vitro study.2022 | duplicate | duplicate | 2022 |
| 599 | Li, JL; Xie, | Long-term remineralizing effect of casein phosphopeptide-amorphous calcium phosphate (CPP-ACP) on early caries lesions in vivo: A systematic review.2014 | duplicate | duplicate | 2014 |
| 600 | Ciribè, M; C | Efficacy of F-ACP-Containing Dental Mousse in the Remineralization of White Spot Lesions after Fixed Orthodontic Therapy: A Randomized Clinical Trial.2024 | title/abstract | irrelevant | 2024 |
| 601 | Suetenkov, D | Photo activated disinfection efficiency of low-intensity laser and comprehensive prevention of caries and gingivitis in adolescents using bracket system.2015 | duplicate | duplicate | 2015 |
| 602 | Rajendran, R | Effect of Remineralization Agents on White Spot Lesions: A Systematic Review.2022 | duplicate | duplicate | 2022 |
| 603 | Singer, L; K | A novel stable biomimetic adhesive coating for functionalization of orthodontic brackets against bacterial colonization and white spot lesions.2025 | fulltext | ineligible intervention | 2025 |
| 604 | Dai, ZX; Liu | Effects of Fluoride and Calcium Phosphate Materials on Remineralization of Mild and Severe White Spot Lesions.2019 | title/abstract | irrelevant | 2019 |
| 605 | Awad, S; El- | EFFECT OF REMINERALIZING AGENTS WITH/WITHOUT LASER IRRADIATION ON ENAMEL PROPERTIES AND SHEAR BOND STRENGTH OF ORTHODONTIC BRACKETS.2022 | title/abstract | irrelevant | 2022 |
| 606 | Ferreira, CJ | Antibacterial and Remineralizing Fillers in Experimental Orthodontic Adhesives.2019 | duplicate | duplicate | 2019 |
| 607 | Brown, ML; D | Ion release from a novel orthodontic resin bonding agent for the reduction and/or prevention of white spot lesions An in vitro study.2011 | duplicate | duplicate | 2011 |
| 608 | Yu, JJ; Qin, | THE EFFECTS OF PREVENTIVE INTERVENTIONS FOR ORTHODONTICALLY INDUCED WHITE SPOT LESIONS: AN UMBRELLA REVIEW AND EVIDENCE MAPPING.2025 | duplicate | duplicate | 2025 |
| 609 | Qin, Q; Yuan | A pH-sensitive, renewable invisible orthodontic aligners coating manipulates antibacterial and in situ remineralization functions to combat enamel demineralization.2024 | duplicate | duplicate | 2024 |
| 610 | Simon, LS; D | Management of Post Orthodontic White Spot Lesions Using Resin Infiltration and CPP-ACP Materials- A Clinical Study.2022 | fulltext | ineligible intervention | 2022 |
| 611 | Zhu, Y; Yan, | The dual anti-caries effect of carboxymethyl chitosan nanogel loaded with chimeric lysin ClyR and amorphous calcium phosphate.2021 | title/abstract | irrelevant | 2021 |
| 612 | Langhorst, S | In vitro remineralization of enamel by polymeric amorphous calcium phosphate composite: Quantitative microradiographic study.2009 | duplicate | duplicate | 2009 |
| 613 | Sezici, YL; | Comparative evaluation of fluoride varnishes, self-assembling peptide-based remineralization agent, and enamel matrix protein derivative on artificial enamel remineralization in vitro.2021 | fulltext | animal study | 2021 |
| 614 | Peng, SY; Gu | Efficacy of peptide-based enamel coatings in the prevention of demineralization using fixed orthodontic brackets in a rat model.2024 | duplicate | duplicate | 2024 |
| 615 | Ahmed, T; Fa | Effect of re-mineralizing surface treatment on the shear bond strength of orthodontic bracket: A systematic review and meta-analysis of in-vitro studies.2023 | duplicate | duplicate | 2023 |
| 616 | Sonesson, M; | Management of post-orthodontic white spot lesions: an updated systematic review.2017 | duplicate | duplicate | 2017 |
| 617 | Wierichs, RJ | Short-term efficacy of caries resin infiltration during treatment with orthodontic fixed appliances. A randomized controlled trial.2023 | fulltext | irrelevant | 2023 |
| 618 | Abbassy, MA; | Characterization of a novel enamel sealer for bioactive remineralization of white spot lesions.2021 | duplicate | duplicate | 2021 |
| 619 | Hamed, S; Ab | Fluoride-amorphous calcium phosphate and biomimetic nano-hydroxyapatite for enamel remineralization: An in-vitro study of surface microhardness and composition.2024 | duplicate | duplicate | 2024 |
| 620 | Xu, L; Zou, | An In Vitro Comparison of Clinpro™ XT and Duraphat Varnish for Protecting Teeth from Discoloration during Orthodontic Treatment.2023 | duplicate | duplicate | 2023 |
| 621 | Pliska, BT; | Treatment of white spot lesions with ACP paste and microabrasion.2012 | title/abstract | irrelevant | 2012 |
| 622 | Samuelraj, S | Does Ozone Enhance the Remineralizing Potential of Nanohydroxyapatite on Artificially Demineralized Enamel? A Laser Induced Fluorescence Study.2014 | title/abstract | irrelevant | 2014 |
| 623 | Songsiriprad | Sodium fluoride mouthrinse used twice daily increased incipient caries lesion remineralization in an in situ model.2014 | duplicate | duplicate | 2014 |
| 624 | Heravi, F; B | Evaluation of Microleakage of Orthodontic Bands Cemented With CPP-ACP-Modified Glass Ionomer Cement.2019 | title/abstract | irrelevant | 2019 |
| 625 | Subramanian, | AN IN VITRO STUDY ON THE EFFICACY OF FOUR REMINERALIZING AGENTS.2022 | duplicate | duplicate | 2022 |
| 626 | Najjaran, H; | Effect of Diode Laser and Fluoride Varnish on Microhardness of enamel: An In Vitro Study.2024 | duplicate | duplicate | 2024 |
| 627 | Loewe, MF; D | Biofilm volume and acidification within initial biofilms formed in situ on buccally and palatally exposed bracket material.2024 | title/abstract | irrelevant | 2024 |
| 628 | Zrinski, MT; | FLUORIDE RELEASE AND RECHARGE POTENTIAL OF REMINERALIZING ORTHODONTIC ADHESIVE SYSTEMS.2019 | title/abstract | irrelevant | 2019 |
| 629 | Heravi, F; B | An in vitro study on the retentive strength of orthodontic bands cemented with CPP-ACP-containing GIC.2016 | duplicate | duplicate | 2016 |
| 630 | Ibrahim, AI; | Enamel conservation orthodontic paradigm via treatment with newly developed remineralizing calcium-phosphate etchant pastes.2025 | title/abstract | irrelevant | 2025 |
| 631 | Kucuk, EB; M | Microcomputed tomography evaluation of white spot lesion remineralization with various procedures.2016 | duplicate | duplicate | 2016 |
| 632 | Chowdhary, S | Antimicrobial Assay of Novel Zirconia and Silver Phyto Nanoparticles Biosynthesized using Ocimum Sanctum and Syzygium Aromaticum Extract-A Preliminary Study.2020 | title/abstract | irrelevant | 2020 |
| 633 | Bhongsatiern | Adjunctive use of fluoride rinsing and brush-on gel increased incipient caries-like lesion remineralization compared with fluoride toothpaste alone in situ.2019 | title/abstract | irrelevant | 2019 |
| 634 | Korkut, B; K | Clinical assessment of demineralization and remineralization surrounding orthodontic brackets with FluoreCam.2017 | duplicate | duplicate | 2017 |
| 635 | Mayne, RJ; C | In-vitro study of the effect of casein phosphopeptide amorphous calcium fluoride phosphate on iatrogenic damage to enamel during orthodontic adhesive removal.2011 | duplicate | duplicate | 2011 |
| 636 | Joshi, S; Va | A Comparative Evaluation of Arginine Complex Combined With Flouride and Two Standard Non-Fluoridated Remineralizing Agents: An In Vitro Study.2024 | title/abstract | irrelevant | 2024 |
| 637 | Hassan, WNW; | A 12-months randomized clinical trial comparing fluoride-based remineralising protocols on post-orthodontic initial caries lesions.2025 | title/abstract | irrelevant | 2025 |
| 638 | Kim, H; Yoo, | A remineralizing orthodontic etchant that utilizes calcium phosphate ion clusters.2022 | title/abstract | irrelevant | 2022 |
| 639 | Strömberg, N | eGenetic- and Lifestyle-dependent Dental Caries Defined by the Acidic Proline-rich Protein Genes PRH1 and PRH2.2017 | title/abstract | irrelevant | 2017 |
| 640 | Philip, N; W | The potential ecological effects of casein phosphopeptide-amorphous calcium phosphate in dental caries prevention.2019 | title/abstract | irrelevant | 2019 |
| 641 | Lei, C; Wang | Biomimetic Self-Maturation Mineralization System for Enamel Repair.2024 | title/abstract | irrelevant | 2024 |
| 642 | Daneshkazemi | Shear bond strength of orthodontic brackets on intact and demineralized enamel after application of resin infiltrant, fluoride varnish and casein phosphopeptide-amorphous calcium phosphate remineralizing agents: in-vitro study.2021 | title/abstract | irrelevant | 2021 |
| 643 | Amato, JN; E | Relation between caries experience and the consumption of sweetened drinks and processed food in children: A population-based study.2023 | title/abstract | irrelevant | 2023 |
| 644 | Shakir, M; M | Innovation of nano-hydrogels loaded with amelogenin peptide and hydroxyapatite nano-particles for remineralisation of artificially induced white spot lesions.2024 | duplicate | duplicate | 2024 |
| 645 | Thierens, LA | The in vitro remineralizing effect of CPP-ACP and CPP-ACPF after 6 and 12 weeks on initial caries lesion.2019 | duplicate | duplicate | 2019 |
| 646 | Zero, DT | Application of clinical models in remineralization research.1999 | title/abstract | irrelevant | 1999 |
| 647 | Hong, SC; Le | Micro-computed tomographic evaluation of the effect of fluoride agents on white spot lesions: An in vitro study.2022 | duplicate | duplicate | 2022 |
| 648 | Demirsoy, KK | The effect of antimicrobial peptide-added adhesive resins on shear bond strength and the adhesive remnant index of orthodontic brackets.2024 | title/abstract | irrelevant | 2024 |
| 649 | Primo, PP; F | Is An Orthodontic Hydrophilic Composite Resistant to Contamination and pH Cycling? In vitro Results.2020 | title/abstract | irrelevant | 2020 |
| 650 | Pretty, IA; | The in vitro detection of early enamel de- and re-mineralization adjacent to bonded orthodontic cleats using quantitative light-induced fluorescence.2003 | duplicate | duplicate | 2003 |
| 651 | Philip, N; L | Casein Phosphopeptide-Amorphous Calcium Phosphate Attenuates Virulence and Modulates Microbial Ecology of Saliva-Derived Polymicrobial Biofilms.2019 | title/abstract | irrelevant | 2019 |
| 652 | Lopes, LB; M | Molar-incisor hypomineralization: an umbrella review.2021 | title/abstract | irrelevant | 2021 |
| 653 | Kecik, D; Ce | Effect of acidulated phosphate fluoride and casein phosphopeptide-amorphous calcium phosphate application on shear bond strength of orthodontic brackets.2008 | title/abstract | irrelevant | 2008 |
| 654 | Giannetti, L | SUPERFICIAL INFILTRATION TO TREAT WHITE HYPOMINERALIZED DEFECTS OF ENAMEL: CLINICAL TRIAL WITH 12-MONTH FOLLOW-UP.2018 | duplicate | duplicate | 2018 |
| 655 | Botelho, J; | Vitamin D Deficiency and Oral Health: A Comprehensive Review.2020 | title/abstract | irrelevant | 2020 |
| 656 | Iijima, M; H | Bracket bond strength and cariostatic potential of an experimental resin adhesive system containing Portland cement.2012 | title/abstract | irrelevant | 2012 |
| 657 | Catros, S; P | Collagen fibrils of human acellular extrinsic fiber cementum.2008 | duplicate | duplicate | 2008 |
| 658 | Abdel Aziz | ASSESSMENT OF SELF-ASSEMBLING PEPTIDE P-11-4 IN THE TREATMENT OF WHITE SPOT LESIONS . | fulltext | included |  |
| 659 |  | Effect of two remineralizing analogues on treatment of post orthodontic enamel white spot lesions using laser fluorescence-based caries detector (an in vivo study) . | fulltext | included |  |
